# Using *Neisseria meningitidis* genomic diversity to inform outbreak strain identification

**DOI:** 10.1371/journal.ppat.1009586

**Published:** 2021-05-18

**Authors:** Adam C. Retchless, Alex Chen, How-Yi Chang, Amy E. Blain, Lucy A. McNamara, Mustapha M. Mustapha, Lee H. Harrison, Xin Wang

**Affiliations:** 1 Division of Bacterial Diseases, National Center for Immunization and Respiratory Diseases, Centers for Disease Control and Prevention, Atlanta, Georgia, United States of America; 2 Microbial Genomic Epidemiology Laboratory, Center for Genomic Epidemiology, University of Pittsburgh, Pittsburgh, Pennsylvania, United States of America; University of Oxford, UNITED KINGDOM

## Abstract

Meningococcal disease is a life-threatening illness caused by the human-restricted bacterium *Neisseria meningitidis*. Outbreaks in the USA involve at least two cases in an organization or community caused by the same serogroup within three months. Genome comparisons, including phylogenetic analysis and quantification of genome distances can provide confirmatory evidence of pathogen transmission during an outbreak. Interpreting genome distances depends on understanding their distribution both among isolates from outbreaks and among those not from outbreaks. Here, we identify outbreak strains based on phylogenetic relationships among 141 *N*. *meningitidis* isolates collected from 28 outbreaks in the USA during 2010–2017 and 1516 non-outbreak isolates collected through contemporaneous meningococcal surveillance. We show that genome distance thresholds based on the maximum SNPs and allele distances among isolates in the phylogenetically defined outbreak strains are sufficient to separate most pairs of non-outbreak isolates into separate strains. Non-outbreak isolate pairs that could not be distinguished from each other based on genetic distances were concentrated in the clonal complexes CC11, CC103, and CC32. Within each of these clonal complexes, phylodynamic analysis identified a group of isolates with extremely low diversity, collected over several years and multiple states. Clusters of isolates with low genetic diversity could indicate increased pathogen transmission, potentially resulting in local outbreaks or nationwide clonal expansions.

## Author summary

Meningococcal disease is a life-threatening illness caused by the bacterium *Neisseria meningitidis*. Meningococcal disease outbreaks occur when the same serogroup of *N*. *meningitidis* causes multiple cases of disease over a short time period in a population such as a community, college, or prison. As with many other pathogens, genome sequencing can reveal genetic relationships among *N*. *meningitidis* based on genomic changes that accumulated as the bacteria were transmitted from person to person. Here, we review 28 outbreaks that occurred over eight years in the United States and identify outbreak strains based on how the *N*. *meningitidis* isolated from these outbreaks relate to each other and to *N*. *meningitidis* isolated from hundreds of other cases from across the country. We show that pairs of isolates from the same outbreak strain have much higher genome similarity than is typical for pairs of isolates that are not from outbreaks; therefore, genome similarity can help delimit outbreak strains during future outbreak investigations. We also identify groups of *N*. *meningitidis* that had similar genomes despite being collected over several years and in multiple states; illustrating how changes in meningococcal disease epidemiology could be affected by the spread of these bacteria.

## Introduction

Meningococcal disease outbreaks in the United States are public health emergencies due to their high case fatality rate [1–3]. CDC guidelines provide flexible thresholds for outbreak declarations, based on detecting multiple primary cases of the same meningococcal serogroup during a 3-month period; outbreaks in organizations may be declared after 2–3 cases, while outbreaks in geographically defined communities require an increased disease incidence [4]. Between 2009 and 2013 in the United States, organization-based outbreaks were most frequently caused by serogroup B, while community-based outbreaks were most frequently caused by serogroup C [2]. Multilocus sequence typing (MLST) places outbreak strains into broad evolutionary lineages called “clonal complexes”, but is not sufficiently discriminatory for differentiating among closely related strains [5,6].

Isolates collected during an outbreak are often clonal, with little genomic diversity, reflecting recent common ancestry [6–8]. Pulsed field gel electrophoresis (PFGE) was conventionally used to differentiate among isolates from outbreaks and other cases based on a quantitative similarity threshold [9]. PFGE is supplanted by whole genome sequence analysis, which provides high-resolution quantification of genome distances [10–12]. Genome sequencing also allows phylogenetic delineation of outbreak strains, based on whether the outbreak isolates form an outbreak-specific clade that includes their most recent common ancestor but excludes other *N*. *meningitidis* that were circulating prior to the outbreak; multiple clades indicate multiple introductions of meningococci into the population, with each clade accumulating diversity as it spreads among asymptomatic carriers [8]. The combination of phylogenetic topology with genome distance metrics has identified outbreak strains in multiple bacterial species, including *Listeria spp*. [13,14], *Legionella pneumophila* [15], and *N*. *meningitidis* [6,7].

The distance between genomes can be quantified as the number of substitutions per site based on maximum likelihood phylogenetic trees; this metric is normalized to genome size and can exclude clustered polymorphisms introduced by recombination. However, other distance metrics such as allele distance based on core genome MLST (cgMLST) and single-nucleotide polymorphisms (SNPs) can be simpler to calculate than recombination-corrected phylogenetic distances, facilitating rapid and standardized processes for distinguishing strains during outbreak investigations. Interpretation of genome distance requires knowledge of the genome distance distribution among the full population of *N*. *meningitidis* isolated from disease cases, which is influenced both by the rate of genome change due to mutation or recombination, and by population changes such as strain introductions or clonal expansions [16,17].

Here we identify genome distance values that indicate outbreaks by evaluating the genomic diversity of meningococcal isolates from US outbreaks relative to the diversity of non-outbreak invasive isolates collected from surveillance programs within the United States and isolates from the UK and Ireland with sequences included in an international genome collection.

## Results

### Genome diversity is structured by geographic and temporal proximity

To understand the overall diversity of meningococcal strains in the United States from 2010 to 2017, we analyzed 1661 genomes of US meningococcal isolates, consisting of 141 isolates from 28 outbreaks, 4 isolates from 2 pairs of cases among close contacts, and 1516 isolates that were neither from a known outbreak nor from close contacts. Contemporaneous meningococcal isolates collected in the UK and Ireland (n = 4091) were included as international comparisons. To facilitate sequence comparisons, we first divided the collection of 5752 genomes into 94 genomic clusters [18], containing from 1 to 1442 genomes, and roughly corresponding to clonal complexes (Adjusted Rand Index of 99.8% for the 5300 genomes with clonal complex assignments; S1 Table). We inferred recombination-corrected maximum likelihood phylogenies for the 32 genomic clusters that contained more than 4 genomes each. The phylogenetic distance among any two isolates in a genomic cluster ranges from the minimum possible value of 2×10^−8^ up to 1.56×10^−3^ subs/site (S1 Fig), and has a strong monotonic association with cgMLST allele distances (Spearman’s rank correlation *r*_s_ = 0.96, range of 0 to 1240 alleles) and SNP distances, both when excluding small SNP clusters (*r*_s_ = 0.94, 0–9067 SNPs, k-mer size k = 25) and when excluding large SNP clusters (*r*_s_ = 0.97, 0–866 SNPs, k = 251) (S2 Fig).

**Fig 1:**
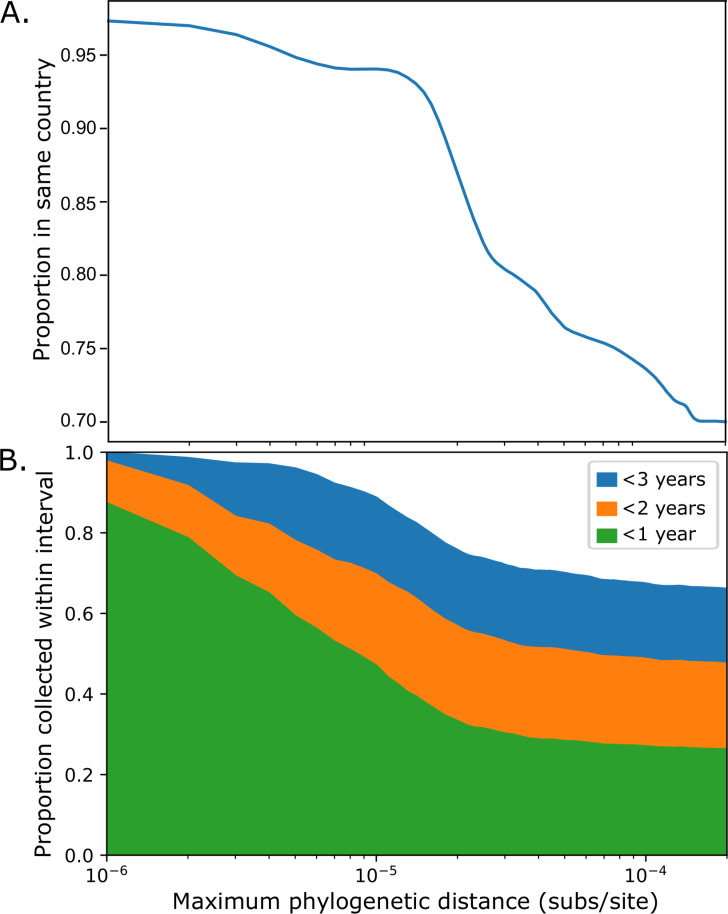
Geographic (A) and temporal (B) *N*. *meningitidis* population structure within the USA. **A**. The proportion of isolate pairs from the same country (USA, UK, or Ireland) are shown as a function of the maximum phylogenetic tree distance between isolates (substitutions per site). **B.** Proportion of isolate pairs collected within 1, 2, or 3 years of each other in the USA, as a function of the maximum phylogenetic tree distance between isolates (substitutions per site). The horizontal scale is identical between the two panels and is shown under panel B.

Isolate pairs with smaller phylogenetic distances between them are more likely to be from the same country (Fig 1A). While 59% of all 2,261,995 pairwise comparisons within genomic clusters are among isolates from the same country, 97% of the 1,235 isolate pairs with fewer than 10^−6^ subs/site between them are from the same country. The proportion within the same country drops rapidly from 94% for the 68,962 isolate pairs that are less than 10^−5^ subs/site apart to 73% for the 1,163,418 isolate pairs that are less than 10^−4^ subs/site apart.

Within the United States, the most similar isolate pairs were collected within short timeframes (Fig 1B). Of the 189,649 comparisons among pairs of US isolates, 25% were between those collected less than 1 year apart, and 65% were between isolates collected less than three years apart; yet for the 676 genome comparisons among US isolates with <10^−6^ subs/site between them, 87% were between isolates collected less than 1 year apart and 99% were among isolates collected less than three years apart.

### Outbreak isolates comprise distinct clades

To characterize the diversification of strains during outbreaks (Table 1), we first evaluated the phylogenetic trees to place isolates from 141 cases from 28 epidemiologically defined outbreaks into “outbreak clades” (Table 2), defined as all isolates that descend from the most recent common ancestor (MRCA) of multiple isolates from each outbreak. Outbreak isolates were excluded from a clade if they were more closely related to an older non-outbreak isolate than to the other isolates from the same outbreak.

**Table 1:** Summary of outbreaks examined in this study.

Outbreak ID	State	Serogroup	Clonal Complex	Isolates analyzed^a^	Year of first isolate	Interval (days)^b^	Clades^c^	Isolates not in a clade
Community-based Outbreaks								
OB02^d^	Oregon	C	CC32	4	2010	149	1	0
OB06^d^	California	B	CC41/44; CC32	3	2011	22	1	1
OB07^d^^,^^e^	New York	C	CC11	13	2011	555	2	2
OB08^d^^,^^e^	California	C	CC11	6	2012	294	1	4
OB09^d^	California	C	CC11	9	2012	189	2	0
OB14^d^^,^^e^	Illinois	C	CC11	10	2015	472	2	0
OB16	Massachusetts	C	CC103	4	2015	738	1	0
OB17	Oregon	C	CC11	3	2011	78	1	0
OB18	Minnesota	B	CC32	2	2013	3	1	0
OB19	California	C	CC11	3	2013	5	1	1
OB21^e^	California	C	CC11	25	2016	472	2	0
OB26^e^	Florida	C	CC11	2	2017	81	1	0
OB27	California	C	CC32	3	2017	117	1	0
Total				87			17	8
Organization-based Outbreaks								
OB03^d^	Ohio	B	CC269	3	2010	21	1	0
OB04^d^	Oklahoma	C	CC11	4	2010	7	1	0
OB05^d^	Colorado	C	CC11	8	2010	245	1	0
OB10^d^^,^^f^	New Jersey	B	CC41/44	8	2013	354	1	0
OB11^d^^,^^f^	California	B	CC32	4	2013	21	1	0
OB12^d^^,^^f^	Oregon	B	CC32	7	2015	113	1	0
OB13^d^^,^^f^	Rhode Island	B	ST-9069^g^	2	2015	21	1	0
OB15^d^	Pennsylvania	B	CC167	2	2011	2	1	0
OB20^f^	California	B	CC32	2	2016	1	1	0
OB22^f^	New Jersey	B	CC11	2	2016	42	1	0
OB23^f^	Wisconsin	B	CC32	3	2016	22	1	0
OB24^f^	Oregon	B	CC32	3	2016	101	1	0
OB25	California	B	CC32	2	2017	4	1	0
OB28	California	B	CC32	2	2017	29	1	0
OB29^d^	Massachusetts	B	CC41/44	2	2017	19	1	0
Total				54			15	0

a. Each isolate is from a different case of meningococcal disease.

b. Number of days between collection of the first and last isolates analyzed from the outbreak.

c. The number of clades on the phylogenetic tree(s) that contained more than one isolate from the outbreak without containing isolates collected more than 6 months before the first outbreak isolate.

d. Outbreaks OB02-OB15 are identified as in Whaley et al. [6].

e. Outbreaks among men who have sex with men (MSM) described by Oliver and Mbaeyi [19].

f. Outbreaks described in Soeters et al. [20].

g. Sequence type (ST) 9069 is not assigned to a clonal complex.

**Table 2:** Summary of outbreak clades identified in the phylogenetic trees.

				Outbreak isolates in clade		Count of non-outbreak isolates in clade					Max. distance between outbreak isolates			
Outbreak ID	Clade ID^a^	Cluster^a^	CC^b^	Count	Interval (days)	Prior^c^	During^d^	Later^e^	Non-US	Days to MRCA^f^	Phylogenetic (subs/site)	Alleles	kSNP (25)	kSNP (251)
**Community-based Outbreak Clades**														
OB02	1	5	CC32	4	149	0	0	0	0	660	4.2×10^−6^	72	459	23
OB06	1	5	CC32	2	22	0	0	0	0	1119	6.7×10^−6^	19	28	19
OB07	1	1	CC11	9	547	1	4	1	2	486	1.1×10^−6^	29	52	33
OB07	2	1	CC11	2	47	0	0	0	0	553	1.8×10^−6^	37	68	25
OB08	1	1	CC11	2	111	0	0	1	0	641	9.4×10^−7^	34	8	28
OB09	1	1	CC11	2	167	0	1	48	0	1887	1.3×10^−5^	56	104	48
OB09	2	1	CC11	7	167	0	1	1	0	1603	9.3×10^−6^	56	262	32
OB14	1	1	CC11	8	288	1	2	1	0	254	3.7×10^−6^	52	226	33
OB14	2	1	CC11	2	164	0	0	0	0	4	2.0×10^−8^	6	7	5
OB16	1	8	CC103	4	738	0	0	2	0	743	4.5×10^−6^	37	191	20
OB17	1	1	CC11	3	78	3	0	0	0	455	9.6×10^−7^	19	48	17
OB18	1	5	CC32	2	3	0	0	1	0	7	3.0×10^−8^	6	24	8
OB19	1	1	CC11	2	2	0	0	0	0	287	2.0×10^−8^	5	13	9
OB21	1	1	CC11	22	337	0	3	0	0	166	3.8×10^−6^	21	101	16
OB21	2	1	CC11	3	87	1	2	3	0	267	2.8×10^−6^	9	24	7
OB26	1	1	CC11	2	81	0	0	1	0	109	4.0×10^−8^	3	4	1
OB27	1	5	CC32	3	117	0	0	0	0	4	3.0×10^−8^	5	10	5
**Totals**				**79**		**6**	**13**	**59**	**2**					
**Organization-based Outbreak Clades**														
OB03	1	4	CC269	3	21	0	0	0	0	1754	5.3×10^−6^	27	74	33
OB04	1	1	CC11	4	7	0	0	0	0	140	9.4×10^−7^	15	7	27
OB05	1	1	CC11	8	245	0	0	0	0	150	9.8×10^−7^	26	24	19
OB10	1	2	CC41/44	8	354	0	2	2	0	ND^g^	4.8×10^−6^	50	48	24
OB11	1	5	CC32	4	21	0	0	0	0	546	3.4×10^−6^	41	250	27
OB12	1	5	CC32	7	113	0	0	22	0	262	2.1×10^−6^	14	69	12
OB13	1	26	ST-9069	2	21	0	0	0	0	ND	1.6×10^−6^	9	29	14
OB15	1	9	CC167	2	2	0	0	0	0	4	2.0×10^−8^	18	6	7
OB20	1	5	CC32	2	1	0	0	0	0	4	8.5×10^−7^	9	24	12
OB22	1	1	CC11	2	42	3	3	2	0	1497	7.0×10^−8^	6	8	2
OB23	1	5	CC32	3	22	0	0	5	0	401	9.4×10^−7^	9	10	8
OB24	1	5	CC32	3	101	0	1	1	0	405	1.7×10^−6^	13	47	12
OB25	1	5	CC32	2	4	6	0	2	0	385	1.0×10^−7^	4	10	9
OB28	1	5	CC32	2	29	1	0	1	0	154	7.0×10^−8^	3	1	4
OB29	1	2	CC41/44	2	19	0	0	0	0	ND	4.7×10^−6^	15	21	21
**Totals**				**54**		**10**	**6**	**35**	**0**					

a. Clades were identified on the phylogenetic tree generated for each genomic cluster; each outbreak clade contains as many isolates as possible from this outbreak without containing any isolates collected more than 6 months before the first isolate from this outbreak.

b. CC: Clonal complex

c. Isolates in the clade collected in the USA prior to the first outbreak isolate.

d. Isolates in the clade collected in the USA after the first isolate in the clade and before the last isolate.

e. Isolates in the clade collected in the USA after the last outbreak isolate. Some clades include isolates from later outbreaks.

f. Number of days between the most recent common ancestor of the clade, and the first outbreak isolate.

g. ND: Not determined; Phylogeny could not be time-calibrated

For each of the 15 organization-based outbreaks, all isolates from a given outbreak belonged to a single outbreak clade. For 6 of 13 community-based outbreaks (OB02, OB16, OB17, OB18, OB26, OB27), all isolates belonged to a single outbreak clade. Another 3 community-based outbreaks (OB09, OB14, OB21) each involved isolates from two clades. The remaining 4 community-based outbreaks (OB06, OB07, OB08, OB19) included some isolates that were not placed into an outbreak clade, while the remaining isolates from the outbreak belonged to one or two outbreak clades. Of the five outbreaks among men who have sex with men [19], four (OB07, OB08, OB14, OB21) had multiple strains. In total, there are 32 outbreak clades for the 28 outbreaks (Tables 1 and 2).

The date of the MRCA was estimated for 29 of the 32 outbreak clades, which were in 5 phylogenetic trees (S3–S7 Figs); the remaining 3 outbreak clades were in trees that could not be calibrated with dates. For the 12 dated clades from organization-based outbreaks, the clade MRCA predated the first isolate by 4–1754 days with a median of 374 days (Table 2). For the 17 clades from community-based outbreaks, the clade MRCA predated the first isolate by 4–1887 days with a median of 455 days (Table 2).

Eighteen outbreak clades included additional isolates from cases that were not epidemiologically linked to the outbreak (Table 2). Seven outbreak clades included non-outbreak isolates that predated the first isolate from the outbreak, but the clades were not divided into smaller outbreak clades due to low bootstrap support (<15%) for any subclades. While three clades included isolates that were collected more than 182 days (6 months) before the first outbreak isolate, the other four clades each included a single isolate that was collected less than 5 months before the first outbreak isolate: 10 days (OB14), 38 days (OB07), 62 days (OB28), and 124 days (OB21). While these prior isolates in the OB14, OB07, and OB28 outbreak clades were collected in the same state as the outbreak isolates (Illinois, New York, and California, respectively), the prior isolate from the OB21 outbreak clade was collected in Nevada while the outbreak occurred in the neighboring state of California.

**Fig 2:**
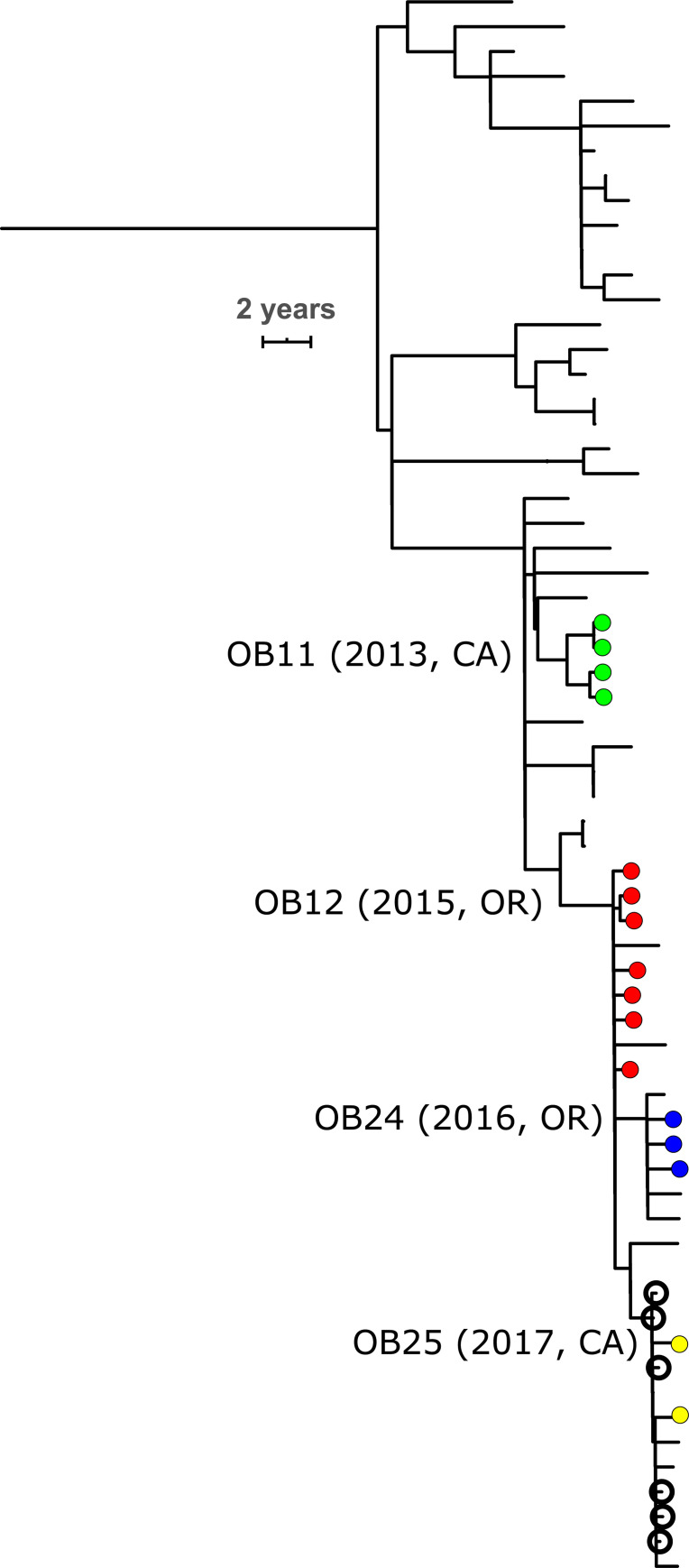
Time-calibrated phylogenetic tree of a CC32 US-specific clade that contains closely related isolates from four organization-based outbreaks (OB11: green; OB12: red; OB24: blue; OB25; yellow). Outbreak isolates are marked with filled circles, and outbreak clades are labeled at their root with the year of the outbreak in parentheses. Open circles indicate isolates collected from cases among high school students attending different schools in the same metropolitan area in California within 5 months of each other. All isolates are serogroup B from the USA. The scale bar is 2 years. The full phylogenetic tree is shown in S5 Fig.

Seventeen outbreak clades (7 organization-based and 10 community-based) included non-outbreak isolates collected during or after the outbreak, while the remaining 15 outbreak clades did not (Table 2). The clades from two CC32 organization-based outbreaks contain the isolates from later organization-based outbreaks in different states. The OB12 outbreak clade (2015, Oregon) includes subclades with all isolates from OB24 (2016, Oregon) and OB25 (2017, California; Fig 2). The OB23 (2016, Wisconsin) outbreak clade includes a subclade with both isolates from OB28 (2017, California). The only non-US isolates in an outbreak clade were two UK isolates collected in 2010 and 2011 that were in an OB07 clade (2011, New York).

### Genome distances as criteria for identifying outbreak strains

**Fig 3:**
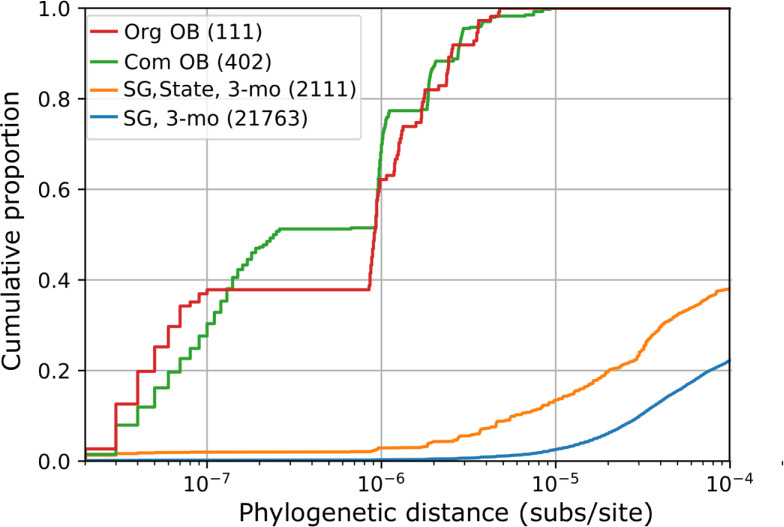
Cumulative distribution of phylogenetic distances (substitutions per site) between pairs of US isolates. Distances are categorized based on whether the isolates were from the same organization-based outbreak clade (Org OB: red), community-based outbreak clade (Com OB: green), or the isolates are not part of a known outbreak, but have the same serogroup and were collected within three months of each other (SG, 3-mo: blue) and from the same state (SG, State, 3-mo: orange). The total number of comparisons in each category is in the parentheses.

To consider how genome diversity can be used to connect isolates belonging to an outbreak strain, we measured the distribution of genome distances between isolates that were from the same outbreak and clade (Table 3), producing 450 pairwise measurements. On the phylogenetic tree, distances ranged from the minimum possible value of 2×10^−8^ up to 1.27×10^−5^ substitutions per site (subs/site), with a median of 8.5×10^−7^ subs/site (interquartile range 7.0×10^−8^–1.6×10^−6^). Distances for other metrics ranged from 0 to 72 cgMLST alleles (median = 10, IQR 6–15), from 0 to 459 SNPs (k = 25, median 11, IQR 7–28), and from 0 to 48 SNPs (k = 251, median 9, IQR 6–13). The greatest distance for each metric was from a community-based outbreak. The maximum phylogenetic tree distances for organization-based outbreaks was less than half of the maximum for community-based outbreaks (5.29×10^−6^ vs. 1.27×10^−5^ subs/site), yet was still the 98^th^ percentile of all distances among pairs of outbreak isolates from the same outbreak clade (Fig 3, other metrics shown in S8 Fig). Comparisons included 11 pairs of isolates that were collected more than 1 year apart (up to 738 days, OB16); however, the greatest distances were observed between isolates collected less than 6 months apart (OB3, OB9) and limiting comparisons to isolate pairs collected less than 6 months part did not substantially change the distribution (S9 Fig).

**Table 3:** Effectiveness of outbreak-based thresholds at distinguishing among non-outbreak isolate pairs with the same serogroup, collected in the same state within 3 months of each other.

	Maximum Distance			Non-outbreak pairs more distant than outbreak maximum^d^		
Distance metric	Org OB^a^	Com OB^b^	Genomic cluster^c^	Org OB max.	Com OB max.	Correlation to tree^e^
**Phylogenetic (subs/site)**	5.29×10^−6^	1.27×10^−5^	1.56×10^−3^	91%	85%	100%
**cgMLST (alleles)**	50	72	1240	82%	81%	96%
**kSNP-25 (SNPs)**	250	459	9067	81%	77%	94%
**kSNP-251 (SNPs)**	33	48	866	85%	83%	97%

a. Organization-based outbreak; maximum distance among isolates from the same outbreak and outbreak clade.

b. Community-based outbreak; maximum distance among isolates from the same outbreak and outbreak clade.

c. Genomic clusters as defined by PopPUNK; roughly equivalent to Clonal Complexes.

d. Percentage is based on 2,022 comparisons among pairs of isolates that have the same serogroup and were collected within 92 days (3 months) of each other in the same state but do not belong to a known outbreak. These 2,022 pairs involved a total of 1,051 isolates.

e. Spearman’s (rank) correlation coefficient for pairwise comparisons within genomic clusters: phylogenetic tree distance vs. each distance metric.

The distribution of genome distances among outbreak strains overlapped with the distribution among the 1516 US isolates that were not from any known outbreak. Of the 1,148,370 pairwise genome distances among these isolates, the minimum was 2×10^−8^ subs/site, while 364 (0.03%) were smaller than 10^−6^ subs/site, 1,103 (0.10%) were smaller than the maximum distance from organization-based outbreaks (5.29×10^−6^ subs/site), and 5,789 (0.50%) were smaller than the maximum distance from any outbreak clade (1.27×10^−5^). The limited overlap between these distributions suggests that the maximum distance from organization-based outbreaks (5.29×10^−6^ subs/site) could be used as threshold for connecting isolates in an outbreak strain, while distinguishing among most isolates that are not from outbreaks.

Of the 1,103 pairs of non-outbreak isolates that would be connected by this threshold, 239 connections (23%) were between isolates that were the same serogroup and were collected within 92 days (3 months) of each other, which are both criteria for outbreak declarations [4]. Of these, 187 were between isolates collected within the same state. An additional 1,835 non-outbreak isolates pairs of the same serogroup and collected within the same state within 92 days of each other were more distant than the threshold. Consequently, this threshold (5.29×10^−6^ subs/site) could distinguish 91% (1,835/2,022) of non-outbreak isolate pairs that were the same serogroup and collected in the same state within 92 days of each other (Fig 3 and Table 3). Other distance metrics could distinguish from 81% to 85% of these 2,022 non-outbreak isolate pairs on the grounds that they were more distant that the maximum distance from organization-based outbreaks (Table 3). The metrics with the strongest rank correlation to the phylogenetic tree distance also had the least overlap of the distribution of distances among non-outbreak isolates and the distribution among outbreak isolates from the same clade (Tables 3 and S2 and S10 Fig).

The genome distance between isolates from different outbreaks was sometimes smaller than the distance between isolates from the same outbreak. The phylogenetic distance threshold (5.29×10^−6^ subs/site) made connections among two group of outbreaks clades. The larger group involved OB11 (2013, CA) and the OB12 (2015, OR) outbreak clade, which includes subclades from OB24 (2016, OR) and OB25 (2017, CA). These outbreak clades are within CC32 (Fig 2). Two of four isolates from the OB11 outbreak clade connected to four of seven isolates from the OB12 outbreak clade (5.10–5.17×10^−6^ subs/site). All isolates from OB22 and OB25 are connected to isolates from the OB12 outbreak clade. Likewise, the CC32 isolates from OB28 (2017, CA) are within the OB23 (2016, WI) outbreak clade and connected to the OB23 isolates.

### Low genetic diversity is concentrated within specific clonal complexes

We next examined whether pairs of highly similar isolates were more common in some clonal complexes than others by examining the average number of phylogenetic distance connections (based on the threshold of 5.29×10^−6^ subs/site) among the 1516 US isolates that were not from any known outbreak, without regard to the collection date. The overall mean number of phylogenetic distance connections per isolate was 1.48 (SD = 3.4), but significantly higher means were obtained for three clonal complexes: CC103 (4.45, SD = 6.7, n = 101, p = 10^−4^ by Monte Carlo subsampling with 9,999 replicates), CC11 (3.07, SD = 4.6, N = 352, p = 10^−4^), and CC32 (1.91, SD = 3.3, n = 99, p = 0.023). Thirty-five percent of the 1516 isolates had a connection to at least one other isolate that was not from a known outbreak; the percentage of isolates with connections was 58% (59/101) for CC103, 59% (208/352) for CC11, 45% (99/219) for CC32, and 20% (166/844) for the remainder of the isolate collection.

We examined whether these differences in diversity among clonal complexes could be explained by different nucleotide substitution rates. Substitution rates were estimated based on the phylogenetic trees for 13 genomic clusters; the median substitution rate was 1.02×10^−6^ subs/site/year (range: 8.5×10^−7^ to 1.58×10^−6^). The rates for cluster 1 (CC11), cluster 5 (CC32), and cluster 8 (CC103) were not outliers, with respective values of 9.0×10^−7^, 9.8×10^−7^, and 1.2×10^−6^ subs/site/year.

The low diversity among isolates of CC11, CC32, and CC103 could reflect recent clonal expansions in the United States that would be evident as clades of US isolates with increasing effective populations sizes [16]. We used the TreeStructure algorithm [16] to partition the time-calibrated phylogenetic trees for each genomic cluster, including both outbreak and non-outbreak isolates, into groups of clades with a similar history of effective population size changes. Cluster 1 (CC11; S3 Fig) contained four partitions, while cluster 5 (CC32; S5 Fig) and cluster 8 (CC103; S6 Fig) each contained three partitions. Both clusters 1 and 5 had one partition that contained only US isolates, while cluster 8 had two partitions with only US isolates; the other partitions included large clades of UK isolates and none had more than 67% US isolates.

The US-specific partition in genomic cluster 1 (CC11) consisted of one clade, which had 36 isolates with a maximum phylogenetic distance of 8.9×10^−6^ subs/site (S3 Fig). This partition contained 34 isolates from California; 22 were collected in 2016–2017 from the community-based outbreak OB21, one from 2013 was considered part of OB08 (as epidemiologically defined) but was not part of an OB08 clade, and 11 non-outbreak California isolates were collected in 2013–2016. The 13 non-outbreak isolates (11 from CA, 2 from other states) in this clade had on average 11.5 (SD = 3.5) connections to other non-outbreak isolates based on the phylogenetic distance threshold.

The US-specific partition in genomic cluster 5 (CC32) also consisted of one clade, which had 63 isolates with a maximum phylogenetic distance of 2.9×10^−5^ subs/site (Figs 2 and S5). The isolates from this partition were collected in six states, primarily in California (n = 37) and Oregon (n = 21), from 2010 to 2017; 15 isolates were from four outbreaks: OB11, OB12, OB24, and OB25 (Fig 2). The 48 non-outbreak isolates had on average 6.3 (SD = 4.6) connections to each other based on the phylogenetic distance threshold. The effective population size of this partition remained constant from 2005 to 2017, while the effective population size of other CC32 partitions decreased (S11 Fig). Notably, the US-specific partition included six isolates that were collected during a period of 145 days (~5 months) from cases among high school students attending different schools in the same metropolitan area in California.

The two US-specific partitions in genomic cluster 8 (CC103) contained 106 of the 111 US isolates, while the third partition included the remaining 5 US isolates and all 24 non-US isolates (S6 Fig). One US partition had 79 isolates with a maximum phylogenetic distance of 7.7×10^−5^ subs/site. These isolates were collected from 16 different states from 2013 to 2017, with 26 from Oregon, 13 from Washington, 13 from Texas, and 6 or fewer from each of the other 13 states. This partition also included all 4 isolates from OB16 in Massachusetts, and one other isolate from that state. In addition, the partition included four isolates from two pairs of cases that occurred among close contacts. The remaining 71 isolates that were not from outbreaks or cases among close contacts had on average 6.0 (SD = 7.4) connections to each other based on the phylogenetic distance threshold. This clade had an increasing effective population size since its estimated origin in 2008 (S12 Fig). The other US-specific partition in genomic cluster 8 contained 27 isolates collected in seven states from 2010 to 2017 with a maximum phylogenetic distance of 6.7×10^−5^ subs/site and an average of 1.0 connections (SD = 1.9) per isolate.

## Discussion

Multiple cases of meningococcal disease caused by the same serogroup within a short time period can indicate an outbreak, prompting public health interventions such as mass vaccination [4]. When isolates are available for whole genome sequencing, genome comparisons can demonstrate that a suspected outbreak isolate does or does not belong to the predominant outbreak strain, thereby aiding efforts to define the population at risk or determine if an outbreak has resolved [4]. In this retrospective study, phylogenetic inference was used to distinguish among strains based on whether outbreak isolates were in the same clades as isolates collected prior to the outbreak [21]. However, during outbreak investigations, genomic data from the relevant pre-outbreak strains may not be available with which to distinguish strains using phylogenetic analysis. Therefore, genome distance metrics such as allele differences and SNPs can supplement other molecular and epidemiologic analyses performed during outbreak investigations by providing rapid and standardizable processes for distinguishing strains.

To establish distance thresholds above which isolate pairs are unlikely to belong to a single outbreak strain, we compiled the genome distances among isolates from the same strain, identified as outbreak-specific clades in a phylogeny. This indicated that 7 of 13 community-based outbreaks involved multiple strains, while organization-based outbreaks always involved a single strain (Tables 1 and 2). Therefore, we considered genome distances from organization-based outbreaks to be the better representative of outbreak strain diversity. Despite the different lineages and serogroups associated with community-based and organization-based outbreaks, the range of genome distances within organization-based outbreak clades was similar to that within community-based outbreak clades (Figs 3 and S8), with the exception of one outlier (OB09) that had exceptionally large genome distances. This community-based outbreak was among California residents with recent travel to (or contact with a traveler to) Tijuana, Mexico where an outbreak occurred in 2012 [22]. OB09 was the only outbreak in this collection that was associated with international travel; the greater diversity of the OB09 outbreak clades may reflect diversity that accumulated in Mexico over several years but was not represented in our US-focused surveillance collection.

The greater the genetic distance between two isolates, the lower the chance that they belong to the same outbreak strain (S8–S10 Figs and S2 Table). The distances between isolates within an outbreak strain may on occasion be greater than the distances measured within the 32 outbreak clades described here, and the decision to include or exclude a case during an outbreak investigation should incorporate all available evidence. In this analysis, the recombination-corrected phylogenetic tree distances were most effective at discriminating among non-outbreak isolates and demonstrating that isolate pairs were not from an outbreak (>5.29×10^−6^ subs/site; Table 3). The simpler kSNP algorithm also discriminated among most pairs of non-outbreak isolates. Discrimination was best when SNPS were identified using long k-mers (251bp) possibly due to the more aggressive removal of SNP clusters that were introduced by homologous recombination and the use of a lower threshold of 33 SNPs (Table 3). This exclusion of potentially recombinant SNPs is achieved at the expense of having fewer SNPs with which to distinguish individual isolates, which is maximized with 25bp k-mers (threshold of 250 SNPs, Table 3). The cgMLST allele distance was also effective at discriminating among non-outbreak isolates. Allele distances have been proposed as a basis for defining genomic groups of isolates within bacterial species [23–25]; our results indicate that a threshold of 50 alleles may be effective at creating genomic groups that correspond to outbreak strains from organizations or geographically defined communities (Table 3). When genomic groups are identified among isolates that are not from outbreaks, they could indicate that rapid transmission of pathogens occurred among a broad population, rather than staying contained within an organization or community where the rapid transmission could produce a noticeable increase of disease incidence. Investigation of these genome-based groups could improve understanding of meningococcal disease transmission. During a nationwide outbreak involving a large number of cases over several years, outbreak strain diversity would likely exceed the thresholds specified above [26].

The phylogenetic analysis also demonstrated that more than half of outbreak clades included isolates that were not considered part of the epidemiologically defined outbreak; two outbreak clades even included isolates from subsequent outbreaks (OB12 with OB24 and OB25; OB23 with OB28). Because outbreak clades were defined to be inclusive, some isolates in the outbreak clade may descend from a close relative of the outbreak isolates, rather than having an ancestor that was transmitted within the population affected by the outbreak. While some of the cases may have had an unrecognized link to the outbreak, other patients may have become infected by a chain of transmission outside of the outbreak-affected population.

The OB12 isolates from Oregon were also similar to the OB11 isolates from California, which were collected over a year earlier and formed a separate clade. This indicates that similar strains were introduced into both of these organizations and caused outbreaks without direct transmission from one organization to the other. Notably, these outbreak isolates were part of a US-specific phylogenetic clade within CC32 (Fig 2) that was distinguished from the remainder of CC32 on the basis that its effective population size remained steady from 2005 to 2017 even as the effective population size of other CC32 clades were decreasing. Many isolates in this clade are closely related to each other, even if they are not involved in outbreaks. Therefore, the similarity between the OB11 and OB12 isolates may reflect the spread of this clade over several years, rather than transmission between these two organizations immediately prior to the outbreaks.

CC11 and CC103 also contained US-specific clades with many isolate pairs connected by small genome distances. The low genomic diversity of isolates in these clades may reflect clonal expansions in the United States. While the CC32 clade contained multiple outbreaks, the CC11 and CC103 clades each contained only one outbreak clade. The CC11 clade consisted primarily of isolates from one outbreak, which may have created the phylodynamic signal leading to the identification of that clade. In contrast, the outbreak caused by CC103 was a small portion of that clade. These clades indicate that even isolates collected several years apart may have small genome distances between them. Based on an estimated substitution rate of 1.02×10^−6^ subs/site/year, it would take on average 5.2 years for an isolate to be distinguished from its direct ancestor using a threshold of 5.29×10^−6^ subs/site. Despite the small genome distances among non-outbreak isolates in CC11, CC32, and CC103, the maximum distances within outbreak strains from these lineages is comparable to the maximum distances within outbreak strains in other lineages, indicating that the same threshold for excluding isolates from outbreaks is appropriate for all *N*. *meningitidis* lineages, but the distance threshold is less effective at confidently distinguishing among strains from these lineages. Strains that are not distinguishable by genome distance may still be distinguishable based on phylogenetic topology, regardless of lineage.

Estimates of the evolutionary time since the MRCA of outbreak strains can be useful for understanding outbreak dynamics [6,27], but reliable estimates cannot be produced for all outbreak strains, limiting the usefulness of this approach during outbreak investigations. While most outbreak clades had an estimated MRCA less than 2 years (730 days) before the first outbreak isolate, others were estimated to be up to five years earlier. This large span of time between the MRCA and the outbreak indicates that the duration of an outbreak is unlikely to contribute substantially to the genomic diversity within an outbreak strain. Due to the low diversity among isolates, the estimate of the time between the MRCA of the outbreak clade and the collection of the isolates could be inflated by artifacts including sequencing errors or the inability to exclude older isolates from the clade. A final factor resulting in early MRCA could be the inclusion of isolates that are actually separate strains; this is a special concern for community-based outbreaks, which are more likely to be multi-clonal.

Outbreaks involving other bacterial pathogens often exhibit fewer differences among isolates (24 SNPs for *Staphylococcus aureus* [28], 7 cgMLST alleles for *Listeria monocytogenes* [14]). The greater divergence among *N*. *meningitidis* isolates may arise from the higher rates of recombination and phase variation in the meningococcal genome [29], or from a prolonged divergence time if the *N*. *meningitidis* strain spreads among asymptomatic carriers prior to the outbreak [8].

Outbreak strains in the United States are evident as clusters of highly similar genomes within the diverse *N*. *meningitidis* population. During outbreak investigations, a genome distance threshold (allele differences or SNPs) can rapidly identify isolates that likely are or are not part of the predominant outbreak strain. In combination with epidemiological data and phylogenetic analysis of other closely related strains, this threshold can help circumscribe the human population within which the outbreak strain is being transmitted. However, pairs of isolates within this distance threshold are not limited to outbreaks, particularly isolates from the CC32 clade that was responsible for four outbreaks during 2013–2017, as well as isolates from the expanding CC103 clade identified in this analysis. Despite the high similarity of invasive disease isolates in these lineages, phylogenetic analysis identified clades associated with each outbreak, thereby excluding most non-outbreak isolates despite their high genomic similarity to the outbreak isolates. Ultimately, while outbreak strains can generally be delimited based solely on genomic comparisons among outbreak isolates, the inclusion of genomic data from population-based surveillance systems enables more precise understanding of pathogen spread both during outbreaks and multi-year clonal expansions.

## Methods

### Ethics statement

This analysis of genomic data was determined not to be human subjects research by the CDC National Center for Immunization and Respiratory Diseases (P_2017_DBD_Wang_411).

### Isolate collection

As part of routine surveillance through the Nationally Notifiable Diseases Surveillance System (NNDSS), jurisdiction health departments are requested to send epidemiological data from meningococcal disease cases to the CDC. NNDSS data were supplemented through Active Bacterial Core surveillance (ABCs) in 2010–2017 [30], expanded surveillance sites in 2013–14 [31], and Enhanced Meningococcal Disease Surveillance (EMDS) in 2015–2017 [32]. This analysis used the genomes of 1661 invasive meningococcal disease isolates collected between 2010 and 2017 as part of ABCs, EMDS, and *ad hoc* submissions from jurisdiction health departments (S1 Table). Based on their epidemiological context, isolates were classified as being from cases in outbreaks (n = 141), cases with known close contact to other cases in the collection (n = 4), or ‘non-outbreak’ cases with no epidemiological links to other cases (n = 1516). The isolates were serogrouped, their genomes sequenced, and MLST loci were identified as previously described [33]. Genome sequences were downloaded from the “MRF Meningococcal Genome Library” (3900 isolates from the UK) and the “Irish Meningococcus Genome Library” (191 isolates from Ireland), hosted by PubMLST on Sept 16, 2019 [34]. MLST sequence types are grouped into clonal complexes in accordance with the PubMLST.org database [34]; not all isolates have complete MLST data, and not all isolates with MLST data are assigned to a clonal complex.

The analysis of outbreak diversity was limited to outbreaks where isolates from two or more cases were available for whole genome sequencing. Of the 28 outbreaks described in this study (Table 1), 14 (OB02-OB15) were described in a previous manuscript analyzing genomic diversity [6], 5 outbreaks (OB20, OB22, OB23, OB24, OB29) were described in a manuscript describing the epidemiology of university-associated serogroup B outbreaks [20], and the remaining 9 outbreaks were identified through routine technical assistance provided by CDC. Outbreaks among men who have sex with men (OB07, OB08, OB14, OB21, OB26) have been reviewed by Oliver and Mbaeyi [19].

### Phylogenetic analysis

Before phylogenetic analysis, the *N*. *meningitidis* isolate collection was partitioned into genomic clusters with lower genomic diversity to avoid long, recombination-saturated branches on the phylogeny. The genomic clusters were identified with PopPunk v1.1 [18], using the easy_run (k = 13) and fit_model (dbscan) algorithms. Genomic clusters roughly correspond to clonal complexes, but also include isolates that were not assigned to clonal complexes (Adjusted Rand Index of 99.8% for the 5300 genomes with clonal complex assignments; S1 Table). For each genomic cluster with five or more isolates, the assemblies were aligned to a single-contig genome assembly using Snippy v4.3.8 [35]; the single-contig genome assemblies were produced from PacBio sequencing as described previously [36], and were selected based on similarity to the genomic cluster and are not necessarily members of the genomic cluster (S1 Table). Positions in the alignment that were missing data from any genome were masked with “N” in all genomes to create a core-genome alignment (ranging from 972 kb to 1,953 kb). Recombinant regions were then masked in each genome alignment using Gubbins v1.4.1 [37], which identifies recombination events by iteratively creating phylogenetic trees and masking regions of the genome where substitutions are over-represented on each branch of the tree, masking 62% to 97% of inferred nucleotide changes on each tree. A final maximum likelihood phylogenetic tree was created from the Gubbins-filtered alignment using RAxML-NG v0.9 [38] using a GTR+G substitution model with a minimum branch length of 10^−8^ substitutions per site, autoMRE bootstopping, and Stamatakis ascertainment correction to account for the removal of monomorphic sites from the Gubbins-filtered alignment. “Outbreak clades” were identified on these phylogenies, defined as all descendants of the most recent common ancestor (MRCA) of the isolates from an outbreak, unless a subset of the outbreak isolates was more closely related to (i.e. formed a subclade with) any isolates that were considered to be outside of the outbreak strain based on being collected more than 182 days (6 months) prior to the first outbreak case. The 6-month threshold is twice the amount of time that can separate the initial cases that justify an outbreak declaration; this longer timespan was chosen because isolates may not be available for the first case in an outbreak.

To perform phylodynamic analysis, the phylogenies were time-calibrated using Treedater v 0.5 [39] with uncorrelated clocks, omega0 = 10^−6^, minblen = 0.01, and temporal constraints. Time-calibration was accepted for 13 of 32 trees that had root-to-tip p-values under 0.01. Demographic differences among clades were identified using TreeStructure v0.1 [16] (p < 0.001 and default parameters otherwise) and population trajectories were inferred using phylodyn v0.9 [40].

### Genome comparison statistics

SNP distances were quantified from alignments generated by kSNP v3.0 [41], which identifies polymorphisms based on nucleotide sequences (k-mers) that vary only at the central site. The optimal k-mer size for distinguishing genomes was determined to be 25 nucleotides using Kchooser [41]. Distances were also calculated using the maximum k-mer size of 251, which excludes SNPs that are separated by 125 nucleotides or fewer. Allele distances between pairs of genomes were quantified as the sum of cgMLST loci that were identified in both genomes with different alleles using BLAST based on the 1605 cgMLST loci defined in the PubMLST database [34]. A locus was considered to be present in the genome assembly if BLAST returned an alignment that included at least 90% of any known allele for that locus. Phylogenetic distances were measured by summing branch lengths on the phylogenetic tree created with RAxML; the phylogenetic analysis algorithms (RAxML and Treedater) require that branch lengths be greater than zero, therefore a minimum length of 1×10^−8^ was selected for these analyses to clearly distinguish branches without substitutions from those that have substitutions, which are expected to be approximately 4.5×10^−7^ subs/site for a branch representing one substitution in a 2.2 Mb genome. Calculations on sequence alignments and phylogenetic trees were performed with BioPython [42] and SciPy [43]. Scripts used to evaluate multiple sequence alignments are available at https://github.com/aretchless/msa_utilities. Monte Carlo subsampling generated a null distribution for the mean number of genome distance connections per isolate using 9,999 simulated samples drawn from the full population of 1516 non-outbreak isolates with the same number of isolates as in each clonal complex.

## Supporting information

S1 Table Information on genome sequences used in this study. The table includes the isolate name, identifier for the genome sequence in the PubMLST database, year collected, country of origin, state of origin (for US isolates), MLST (Sequence Type and Clonal Complex), finetyping antigens (PorA, FetA), serogroup, PopPunk genomic cluster, outbreak identifier if applicable, and whether it was a single-contig genome assembly used as a reference for alignment of the genomic cluster.
(XLSX)

S2 Table Threshold values for each distance metric based on percentile of comparisons within outbreak isolates from the same clade. Values correspond to the ROC curves in S10 Fig, with “False Positive” defined as comparisons with outbreaks exceeding the threshold, and “True Positive” defined as comparisons among non-outbreak isolates exceeding the threshold.
(XLSX)

S1 Fig Violin plot showing the distribution of phylogenetic distances within genomic clusters. The 22 genomic clusters with ten or more genomes are labeled with their numeric identifier, while the 10 clusters with five to nine genomes are grouped together as “Other”. Phylogenetic trees were not inferred for the 62 genomic clusters with four or fewer genomes in them (including 43 singleton ‘clusters’). Substitutions per site between pairs of genomes were calculated as the sum of branch lengths separating the two genomes on a recombination-corrected maximum likelihood phylogenetic tree that was inferred for each cluster.
(DOCX)

S2 Fig Relationship of phylogenetic distance to other genome distance metrics. The scatter plots on the left (A, C, E) show the full range of distances within genomic clusters and the Spearman rank correlation (*r*_s_), while the scatter plots on the right (B, D, F) are limited to closely related isolate pairs, up to a phylogenetic distance of 10^−4^ subs/site. The distance metrics include SNP distances identified with 25bp k-mer (A, B), SNP distances identified with a 251bp k-mer (C,D), and cgMLST allele distances (E,F).
(DOCX)

S3 Fig Time-calibrated phylogeny of genomic cluster 1 (CC11, 1442 isolates, 1,160,070bp core genome alignment). The inner ring shows the country of origin and the outer ring shows serogroup (missing data is uncolored). Internal shading shows TreeStructure partitions, with the US-specific partition shaded red. Black dots on branch tips indicate isolates from 14 outbreak clades in the USA. Tree scale bar is 10 years. The estimated evolutionary rate is 9.0×10^−7^ subs/site/year.
(DOCX)

S4 Fig Time-calibrated phylogeny of genomic cluster 4 (CC269, 712 isolates, 1,045,137bp core genome alignment). Inner ring shows the country of origin, outer ring shows serogroup. Black dots on leaf tips indicate isolates from one outbreak clade in the USA. Tree scale bar is 10 years. The estimated evolutionary rate is 8.6×10^−7^ subs/site/year.
(DOCX)

S5 Fig Time-calibrated phylogeny of genomic cluster 5 (CC32, 466 isolates, 1,277,833bp core genome alignment). Inner ring shows the country of origin, outer ring shows serogroup. Internal shading shows TreeStructure partitions, with the US-specific partition #1 shaded red (detail in Fig 2). Black dots indicate isolates from 11 outbreak clades in the USA. Tree scale bar is 10 years. The estimated evolutionary rate is 9.8×10^−7^ subs/site/year.
(DOCX)

S6 Fig Time-calibrated phylogeny of genomic cluster 8 (CC103, 140 isolates, 1,597,249bp core genome alignment). Inner ring shows the country of origin, outer ring shows serogroup. Internal shading shows TreeStructure partitions: red, partition 1; green, partition 2; blue, partition 3. Black dots indicate isolates from one outbreak clade in the USA. Tree scale bar is 10 years. The estimated evolutionary rate is 1.2×10^−6^ subs/site/year.
(DOCX)

S7 Fig Time-calibrated phylogeny of genomic cluster 9 (CC167, 105 isolates, 1,543,098bp core genome alignment). Inner ring shows the country of origin, outer ring shows serogroup. Black dots indicate isolates from one outbreak clade in the USA. Tree scale bar is 10 years. The estimated evolutionary rate is 1.0×10^−6^ subs/site/year.
(DOCX)

S8 Fig Cumulative distribution of alternative distance metrics: kSNP-25 (A), kSNP-251 (B), cgMLST (C). As in Fig 2, distances are categorized based on whether the isolates were collected from the same organization-based outbreak (Org OB: red), community-based outbreak (Com OB: green), or the isolates are not part of a known outbreak, but have the same serogroup and were collected within three months of each other (SG, 3-mo: blue) and from the same state (SG, State, 3-mo: orange). The total number of comparisons in each category is in the parentheses.
(DOCX)

S9 Fig Cumulative distribution of distances among outbreak isolates in the same clade, including a subset that was limited to the pairs that were collected within 6 months of each other. As in Figs 3 and S8 distances are categorized based on whether the isolates were collected from the same organization-based outbreak (Org OB: red), or community-based outbreak (Com OB: green); lines have been added for all outbreaks (black) and comparisons within 6 months (brown).
(DOCX)

S10 Fig ROC (receiver operating characteristic) curves for distinguishing isolate pairs based on each distance metric. AUC (area under the curve) is reported in the legend. A ‘positive’ result occurs when the distance between the two genomes is greater than the threshold value, which decreases as ROC curves move to the top-right. A true positive occurs among the 2,022 pairs of non-outbreak isolates that were the same serogroup and collected in the same state within 92 days of each other; a false positive occurs among the 452 pairs of outbreak isolates that were in the same outbreak clade. The lowest TPR value shown on the vertical axis is 75%; all TPR values were at least 77% (Table 3) while the FPR was 0%. Threshold values for several FPRs are shown in S2 Table.
(DOCX)

S11 Fig Demographic history of genomic cluster 5 partitions (CC32, shown in S5 Fig). Time is measured in years before 2017, and the effective population size is scaled to the number of generations per year. **A**. Partition 1, consisting of a single clade of 62 US isolates indicated by red shading in S5 Fig. **B**. Partition 2 (blue shading). **C**. Partition 3 (green shading).
(DOCX)

S12 Fig Demographic history of cluster 8 partitions (CC103, shown in S6 Fig). Time is measured in years before 2017, and the effective population size is scaled to the number of generations per year. **A**. Partition 1 consisting of 5 US isolates and 24 UK isolates (red shading in S6 Fig). **B**. Partition 2, consisting of 27 US isolates (green shading). **C**. Partition 3, consisting of 79 US isolates (blue shading).
(DOCX)

## References

[1] MacNeil JR, Blain AE, Wang X, Cohn AC. Current Epidemiology and Trends in Meningococcal Disease-United States, 1996–2015. Clin Infect Dis. 2018;66(8):1276–81. Epub 2017/11/11. doi: 10.1093/cid/cix993 . 29126310

[2] Mbaeyi SA, Blain A, Whaley MJ, Wang X, Cohn AC, MacNeil JR. Epidemiology of meningococcal disease outbreaks in the United States, 2009–2013. Clin Infect Dis. 2018;68(4):580–5. Epub 2018/07/10. doi: 10.1093/cid/ciy548 . 29982382

[3] Trotter C, Ramsay M, Harrison L. Introduction and epidemiology of meningococcal disease. In: Feavers I, Pollard AJ, Sadarangani M, editors. Handbook of Meningococcal Disease Management. Cham: Springer International Publishing; 2016. p. 1–14.

[4] Centers for Disease Control and Prevention. Guidance for the evaluation and public health management of suspected outbreaks of meningococcal disease. (version 2.0) 2019 [cited 2020 May 5]. Available from: https://www.cdc.gov/meningococcal/downloads/meningococcal-outbreak-guidance.pdf.

[5] Jolley KA, Brehony C, Maiden MC. Molecular typing of meningococci: recommendations for target choice and nomenclature. FEMS microbiology reviews. 2007;31(1):89–96. Epub 2006/12/16. doi: 10.1111/j.1574-6976.2006.00057.x . 17168996

[6] Whaley MJ, Joseph SJ, Retchless AC, Kretz CB, Blain A, Hu F, et al. Whole genome sequencing for investigations of meningococcal outbreaks in the United States: a retrospective analysis. Scientific reports. 2018;8(1):15803. Epub 2018/10/27. doi: 10.1038/s41598-018-33622-5 ; PubMed Central PMCID: PMC6202316. 30361650 PMC6202316

[7] Saltykova A, Mattheus W, Bertrand S, Roosens NHC, Marchal K, De Keersmaecker SCJ. Detailed Evaluation of Data Analysis Tools for Subtyping of Bacterial Isolates Based on Whole Genome Sequencing: *Neisseria meningitidis* as a Proof of Concept. Frontiers in microbiology. 2019;10:2897. Epub 2020/01/11. doi: 10.3389/fmicb.2019.02897 ; PubMed Central PMCID: PMC6930190. 31921072 PMC6930190

[8] Holmes JC, Green LR, Oldfield NJ, Turner DPJ, Bayliss CD. Rapid Transmission of a Hyper-Virulent Meningococcal Clone Due to High Effective Contact Numbers and Super Spreaders. Front Genet. 2020;11:579411. Epub 2020/12/29. doi: 10.3389/fgene.2020.579411 ; PubMed Central PMCID: PMC7750637. 33365047 PMC7750637

[9] Popovic T, Schmink S, Rosenstein NA, Ajello GW, Reeves MW, Plikaytis B, et al. Evaluation of pulsed-field gel electrophoresis in epidemiological investigations of meningococcal disease outbreaks caused by *Neisseria meningitidis* serogroup C. J Clin Microbiol. 2001;39(1):75–85. Epub 2001/01/04. doi: 10.1128/JCM.39.1.75-85.2001 ; PubMed Central PMCID: PMC87683. 11136752 PMC87683

[10] Ezeoke I, Galac MR, Lin Y, Liem AT, Roth PA, Kilianski A, et al. Tracking a serial killer: Integrating phylogenetic relationships, epidemiology, and geography for two invasive meningococcal disease outbreaks. PLoS One. 2018;13(11):e0202615. Epub 2018/11/30. doi: 10.1371/journal.pone.0202615 ; PubMed Central PMCID: PMC6261407. 30485280 PMC6261407

[11] Stefanelli P, Fazio C, Vacca P, Palmieri A, Ambrosio L, Neri A, et al. An outbreak of severe invasive meningococcal disease due to a capsular switched *Neisseria meningitidis* hypervirulent strain B:cc11. Clinical microbiology and infection: the official publication of the European Society of Clinical Microbiology and Infectious Diseases. 2019;25(1):111.e1–.e4. Epub 2018/07/24. doi: 10.1016/j.cmi.2018.07.014 . 30036673

[12] Lawler J, Lucidarme J, Parikh S, Smith L, Campbell H, Borrow R, et al. Suspected cluster of *Neisseria meningitidis* W invasive disease in an elderly care home: do new laboratory methods aid public health action? United Kingdom, 2015. Euro surveillance: bulletin Europeen sur les maladies transmissibles = European communicable disease bulletin. 2019;24(23). Epub 2019/06/13. doi: 10.2807/1560-7917.Es.2019.24.23.1900070 ; PubMed Central PMCID: PMC6561014. 31186079 PMC6561014

[13] Pightling AW, Pettengill JB, Luo Y, Baugher JD, Rand H, Strain E. Interpreting Whole-Genome Sequence Analyses of Foodborne Bacteria for Regulatory Applications and Outbreak Investigations. Frontiers in microbiology. 2018;9:1482. Epub 2018/07/26. doi: 10.3389/fmicb.2018.01482 ; PubMed Central PMCID: PMC6048267. 30042741 PMC6048267

[14] Moura A, Criscuolo A, Pouseele H, Maury MM, Leclercq A, Tarr C, et al. Whole genome-based population biology and epidemiological surveillance of *Listeria monocytogenes*. Nature microbiology. 2016;2:16185. Epub 2016/10/11. doi: 10.1038/nmicrobiol.2016.185 . 27723724 PMC8903085

[15] Raphael BH, Baker DJ, Nazarian E, Lapierre P, Bopp D, Kozak-Muiznieks NA, et al. Genomic Resolution of Outbreak-Associated *Legionella pneumophila* Serogroup 1 Isolates from New York State. Applied and environmental microbiology. 2016;82(12):3582–90. Epub 2016/04/10. doi: 10.1128/AEM.00362-16 ; PubMed Central PMCID: PMC4959152. 27060122 PMC4959152

[16] Volz EM, Wiuf C, Grad YH, Frost SDW, Dennis AM, Didelot X. Identification of hidden population structure in time-scaled phylogenies. Systematic biology. 2020;69(5):884–96. Epub 2020/02/13. doi: 10.1093/sysbio/syaa009 . 32049340 PMC8559910

[17] Mustapha MM, Marsh JW, Shutt KA, Schlackman J, Ezeonwuka C, Farley MM, et al. Transmission Dynamics and Microevolution of *Neisseria meningitidis* During Carriage and Invasive Disease in High School Students in Georgia and Maryland, 2006–2007. The Journal of infectious diseases. 2020. doi: 10.1093/infdis/jiaa674 33107578 PMC8205623

[18] Lees JA, Harris SR, Tonkin-Hill G, Gladstone RA, Lo SW, Weiser JN, et al. Fast and flexible bacterial genomic epidemiology with PopPUNK. Genome Res. 2019;29(2):304–16. Epub 2019/01/27. doi: 10.1101/gr.241455.118 ; PubMed Central PMCID: PMC6360808. 30679308 PMC6360808

[19] Oliver SE, Mbaeyi SA. A Review of Global Epidemiology and Response to Meningococcal Disease Outbreaks among Men Who Have Sex with Men, 2001–2018. Current Epidemiology Reports. 2018;5(4):321–30. doi: 10.1007/s40471-018-0170-z

[20] Soeters HM, McNamara LA, Blain AE, Whaley M, MacNeil JR, Hariri S, et al. University-Based Outbreaks of Meningococcal Disease Caused by Serogroup B, United States, 2013–2018. Emerging infectious diseases. 2019;25(3):434–40. Epub 2019/02/23. doi: 10.3201/eid2503.181574 ; PubMed Central PMCID: PMC6390773. 30789140 PMC6390773

[21] Villabona-Arenas CJ, Hanage WP, Tully DC. Phylogenetic interpretation during outbreaks requires caution. Nature microbiology. 2020;5(7):876–7. Epub 2020/05/20. doi: 10.1038/s41564-020-0738-5 . 32427978 PMC8168400

[22] Chacon-Cruz E, Espinosa-De Los Monteros LE, Navarro-Alvarez S, Aranda-Lozano JL, Volker-Soberanes ML, Rivas-Landeros RM, et al. An outbreak of serogroup C (ST-11) meningococcal disease in Tijuana, Mexico. Therapeutic advances in vaccines. 2014;2(3):71–6. Epub 2014/05/03. doi: 10.1177/2051013614526592 ; PubMed Central PMCID: PMC3991157. 24790731 PMC3991157

[23] Cody AJ, Bray JE, Jolley KA, McCarthy ND, Maiden MCJ. Core Genome Multilocus Sequence Typing Scheme for Stable, Comparative Analyses of *Campylobacter jejuni* and *C*. *coli* Human Disease Isolates. J Clin Microbiol. 2017;55(7):2086–97. Epub 2017/04/28. doi: 10.1128/JCM.00080-17 J Journal of Clinical Microbiology. ; PubMed Central PMCID: PMC5483910. 28446571 PMC5483910

[24] Harrison OB, Cehovin A, Skett J, Jolley KA, Massari P, Genco CA, et al. *Neisseria gonorrhoeae* Population Genomics: Use of the Gonococcal Core Genome to Improve Surveillance of Antimicrobial Resistance. The Journal of infectious diseases. 2020;222(11):1816–25. Epub 2020/03/13. doi: 10.1093/infdis/jiaa002 ; PubMed Central PMCID: PMC7653085. 32163580 PMC7653085

[25] Jamet A, Guglielmini J, Brancotte B, Coureuil M, Euphrasie D, Meyer J, et al. High resolution typing of *Staphylococcus epidermidis* based on cgMLST to investigate the hospital spread of multidrug resistant clones. J Clin Microbiol. 2020. Epub 2020/12/18. doi: 10.1128/jcm.02454-20 . 33328176 PMC8106705

[26] Lucidarme J, Scott KJ, Ure R, Smith A, Lindsay D, Stenmark B, et al. An international invasive meningococcal disease outbreak due to a novel and rapidly expanding serogroup W strain, Scotland and Sweden, July to August 2015. Euro surveillance: bulletin Europeen sur les maladies transmissibles = European communicable disease bulletin. 2016;21(45). Epub 2016/12/06. doi: 10.2807/1560-7917.Es.2016.21.45.30395 ; PubMed Central PMCID: PMC5144941. 27918265 PMC5144941

[27] Lo Presti A, Neri A, Fazio C, Vacca P, Ambrosio L, Grazian C, et al. Reconstruction of Dispersal Patterns of Hypervirulent Meningococcal Strains of Serogroup C:cc11 by Phylogenomic Time Trees. J Clin Microbiol. 2019;58(1). Epub 2019/11/02. doi: 10.1128/jcm.01351-19 ; PubMed Central PMCID: PMC6935922. 31666361 PMC6935922

[28] Coll F, Raven KE, Knight GM, Blane B, Harrison EM, Leek D, et al. Definition of a genetic relatedness cutoff to exclude recent transmission of meticillin-resistant *Staphylococcus aureus*: a genomic epidemiology analysis. The Lancet Microbe. 2020;1(8):e328–e35. Epub 2020/12/15. doi: 10.1016/S2666-5247(20)30149-X ; PubMed Central PMCID: PMC7721685. 33313577 PMC7721685

[29] Caugant DA, Brynildsrud OB. *Neisseria meningitidis*: using genomics to understand diversity, evolution and pathogenesis. Nature Reviews Microbiology. 2020;18(2):84–96. doi: 10.1038/s41579-019-0282-6 31705134

[30] Langley G, Schaffner W, Farley MM, Lynfield R, Bennett NM, Reingold A, et al. Twenty Years of Active Bacterial Core Surveillance. Emerging infectious diseases. 2015;21(9):1520–8. Epub 2015/08/21. doi: 10.3201/eid2109.141333 ; PubMed Central PMCID: PMC4550139. 26292067 PMC4550139

[31] Chang HY, Vuong J, Hu F, Liberator P, Chen A, Kretz CB, et al. Distribution of *Neisseria meningitidis* serogroup b (NmB) vaccine antigens in meningococcal disease causing isolates in the United States during 2009–2014, prior to NmB vaccine licensure. The Journal of infection. 2019;79(5):426–34. Epub 2019/09/11. doi: 10.1016/j.jinf.2019.09.001 . 31505201

[32] Enhanced Meningococcal Disease Surveillance Report, 2017: Centers for Disease Control and Prevention; 2018 [cited 2020 November 2]. Available from: https://www.cdc.gov/meningococcal/downloads/NCIRD-EMS-Report-2017.pdf.

[33] Potts CC, Joseph SJ, Chang HY, Chen A, Vuong J, Hu F, et al. Population structure of invasive Neisseria meningitidis in the United States, 2011–15. The Journal of infection. 2018;77(5):427–34. Epub 2018/07/03. doi: 10.1016/j.jinf.2018.06.008 ; PubMed Central PMCID: PMC6485409. 29964139 PMC6485409

[34] Jolley KA, Bray JE, Maiden MCJ. Open-access bacterial population genomics: BIGSdb software, the PubMLST.org website and their applications. Wellcome open research. 2018;3:124–. Epub 2018/10/23. doi: 10.12688/wellcomeopenres.14826.1 ; PubMed Central PMCID: PMC6192448. 30345391 PMC6192448

[35] Seemann T. Snippy: Rapid haploid variant calling and core SNP phylogeny 2015. Available from: https://github.com/tseemann/snippy.

[36] Kretz CB, Retchless AC, Sidikou F, Issaka B, Ousmane S, Schwartz S, et al. Whole-Genome Characterization of Epidemic *Neisseria meningitidis* Serogroup C and Resurgence of Serogroup W, Niger, 2015. Emerging infectious diseases. 2016;22(10):1762–8. Epub 2016/09/21. doi: 10.3201/eid2210.160468 ; PubMed Central PMCID: PMC5038424. 27649262 PMC5038424

[37] Croucher NJ, Page AJ, Connor TR, Delaney AJ, Keane JA, Bentley SD, et al. Rapid phylogenetic analysis of large samples of recombinant bacterial whole genome sequences using Gubbins. Nucleic acids research. 2015;43(3):e15. Epub 2014/11/22. doi: 10.1093/nar/gku1196 ; PubMed Central PMCID: PMC4330336. 25414349 PMC4330336

[38] Kozlov AM, Darriba D, Flouri T, Morel B, Stamatakis A. RAxML-NG: a fast, scalable and user-friendly tool for maximum likelihood phylogenetic inference. Bioinformatics (Oxford, England). 2019;35(21):4453–5. Epub 2019/05/10. doi: 10.1093/bioinformatics/btz305 ; PubMed Central PMCID: PMC6821337. 31070718 PMC6821337

[39] Volz EM, Frost SD. Sampling through time and phylodynamic inference with coalescent and birth-death models. Journal of the Royal Society, Interface. 2014;11(101):20140945. Epub 2014/11/18. doi: 10.1098/rsif.2014.0945 ; PubMed Central PMCID: PMC4223917. 25401173 PMC4223917

[40] Karcher MD, Palacios JA, Lan S, Minin VN. phylodyn: an R package for phylodynamic simulation and inference. Molecular ecology resources. 2017;17(1):96–100. Epub 2016/11/02. doi: 10.1111/1755-0998.12630 ; PubMed Central PMCID: PMC5466693. 27801980 PMC5466693

[41] Gardner SN, Slezak T, Hall BG. kSNP3.0: SNP detection and phylogenetic analysis of genomes without genome alignment or reference genome. Bioinformatics (Oxford, England). 2015;31(17):2877–8. Epub 2015/04/29. doi: 10.1093/bioinformatics/btv271 . 25913206

[42] Cock PJ, Antao T, Chang JT, Chapman BA, Cox CJ, Dalke A, et al. Biopython: freely available Python tools for computational molecular biology and bioinformatics. Bioinformatics (Oxford, England). 2009;25(11):1422–3. Epub 2009/03/24. doi: 10.1093/bioinformatics/btp163 ; PubMed Central PMCID: PMC2682512. 19304878 PMC2682512

[43] Virtanen P, Gommers R, Oliphant TE, Haberland M, Reddy T, Cournapeau D, et al. SciPy 1.0: fundamental algorithms for scientific computing in Python. Nat Methods. 2020;17(3):261–72. Epub 2020/02/06. doi: 10.1038/s41592-019-0686-2 ; PubMed Central PMCID: PMC7056644. 32015543 PMC7056644

