# Supplementary material for: Using Neisseria meningitidis genomic diversity to inform outbreak strain identification
Source: PLoS Pathog. 2021 May 18;17(5):e1009586. doi: 10.1371/journal.ppat.1009586 (PMC8177650; doi:10.1371/journal.ppat.1009586)
Supplement: S1 Fig — The 22 genomic clusters with ten or more genomes are labeled with their numeric identifier, while the 10 clusters with five to nine genomes are grouped together as “Other”. Phylogenetic trees were not inferred for the 62 genomic clusters with four or fewer genomes in them (including 43 singleton ‘clusters’). Substitutions per site between pairs of genomes were calculated as the sum of branch lengths separating the two genomes on a recombination-corrected maximum likelihood phylogenetic tree that was inferred for each cluster. (DOCX) [file ppat.1009586.s003.docx]

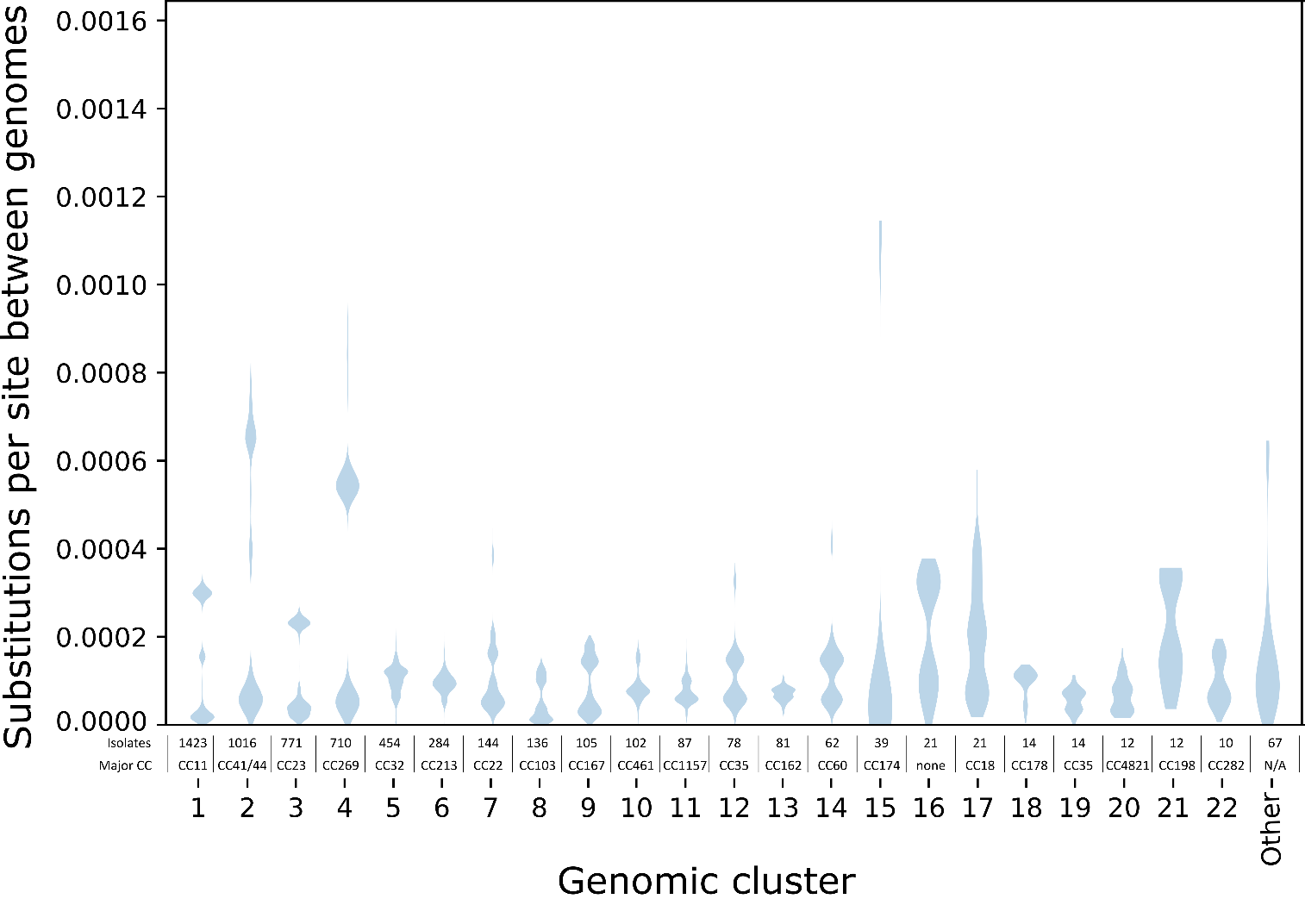


**S1 Fig:** Violin plot showing the distribution of phylogenetic distances within genomic clusters. The 22 genomic clusters with ten or more genomes are labeled with their numeric identifier, while the 10 clusters with five to nine genomes are grouped together as “Other”. The major clonal complexes (CC) and number of isolates in each cluster is written above the numeric identifier. The genomes in cluster 16 had sequence types that were not assigned to any clonal complex (labeled ‘none’), and the major clonal complex was not applicable (“N/A”) to the ‘other’ category because it comprised multiple clusters. Substitutions per site between pairs of genomes were calculated as the sum of branch lengths separating the two genomes on a recombination-corrected maximum likelihood phylogenetic tree that was inferred for each cluster. Phylogenetic trees were not inferred for the 62 genomic clusters with four or fewer genomes in them (including 43 singleton ‘clusters’).
