# Supplementary material for: Using Neisseria meningitidis genomic diversity to inform outbreak strain identification
Source: PLoS Pathog. 2021 May 18;17(5):e1009586. doi: 10.1371/journal.ppat.1009586 (PMC8177650; doi:10.1371/journal.ppat.1009586)
Supplement: S2 Fig — The scatter plots on the left (A, C, E) show the full range of distances within genomic clusters and the Spearman rank correlation (rs), while the scatter plots on the right (B, D, F) are limited to closely related isolate pairs, up to a phylogenetic distance of 10−4 subs/site. The distance metrics include SNP distances identified with 25bp k-mer (A, B), SNP distances identified with a 251bp k-mer (C,D), and cgMLST allele distances (E,F). (DOCX) [file ppat.1009586.s004.docx]

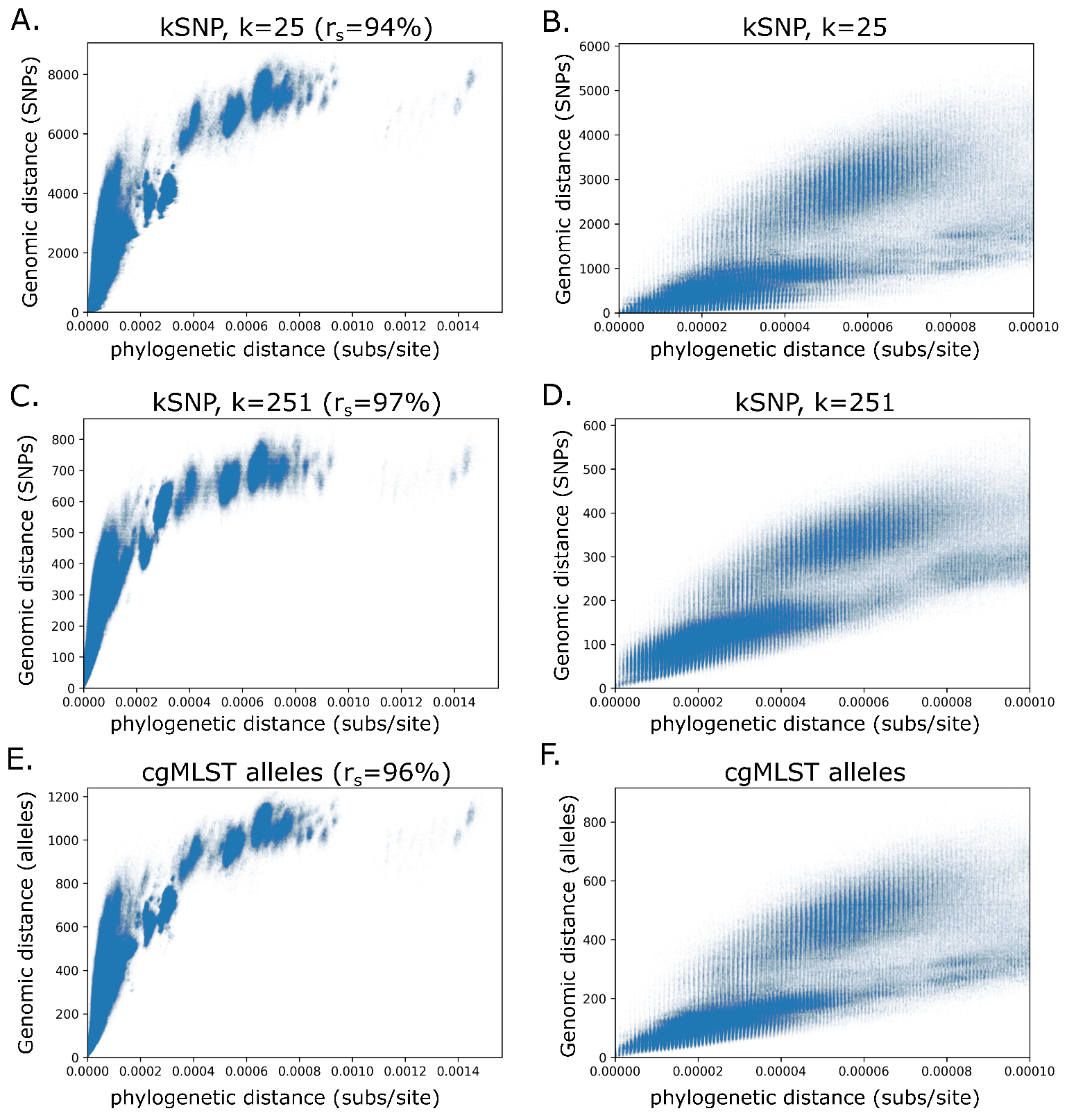


**S2 Fig**: Relationship of phylogenetic distance to other genome distance metrics. The scatter plots on the left (A, C, E) show the full range of distances within genomic clusters and the Spearman rank correlation (*r*_s_), while the scatter plots on the right (B, D, F) are limited to closely related isolate pairs, up to a phylogenetic distance of 10^-4^ subs/site. The distance metrics include SNP distances identified with 25bp k-mer (A, B), SNP distances identified with a 251bp k-mer (C,D), and cgMLST allele distances (E,F).
