# Supplementary material for: Using Neisseria meningitidis genomic diversity to inform outbreak strain identification
Source: PLoS Pathog. 2021 May 18;17(5):e1009586. doi: 10.1371/journal.ppat.1009586 (PMC8177650; doi:10.1371/journal.ppat.1009586)
Supplement: S3 Fig — The inner ring shows the country of origin and the outer ring shows serogroup (missing data is uncolored). Internal shading shows TreeStructure partitions, with the US-specific partition shaded red. Black dots on branch tips indicate isolates from 14 outbreak clades in the USA. Tree scale bar is 10 years. The estimated evolutionary rate is 9.0×10^−7^ subs/site/year. (DOCX) [file ppat.1009586.s005.docx]

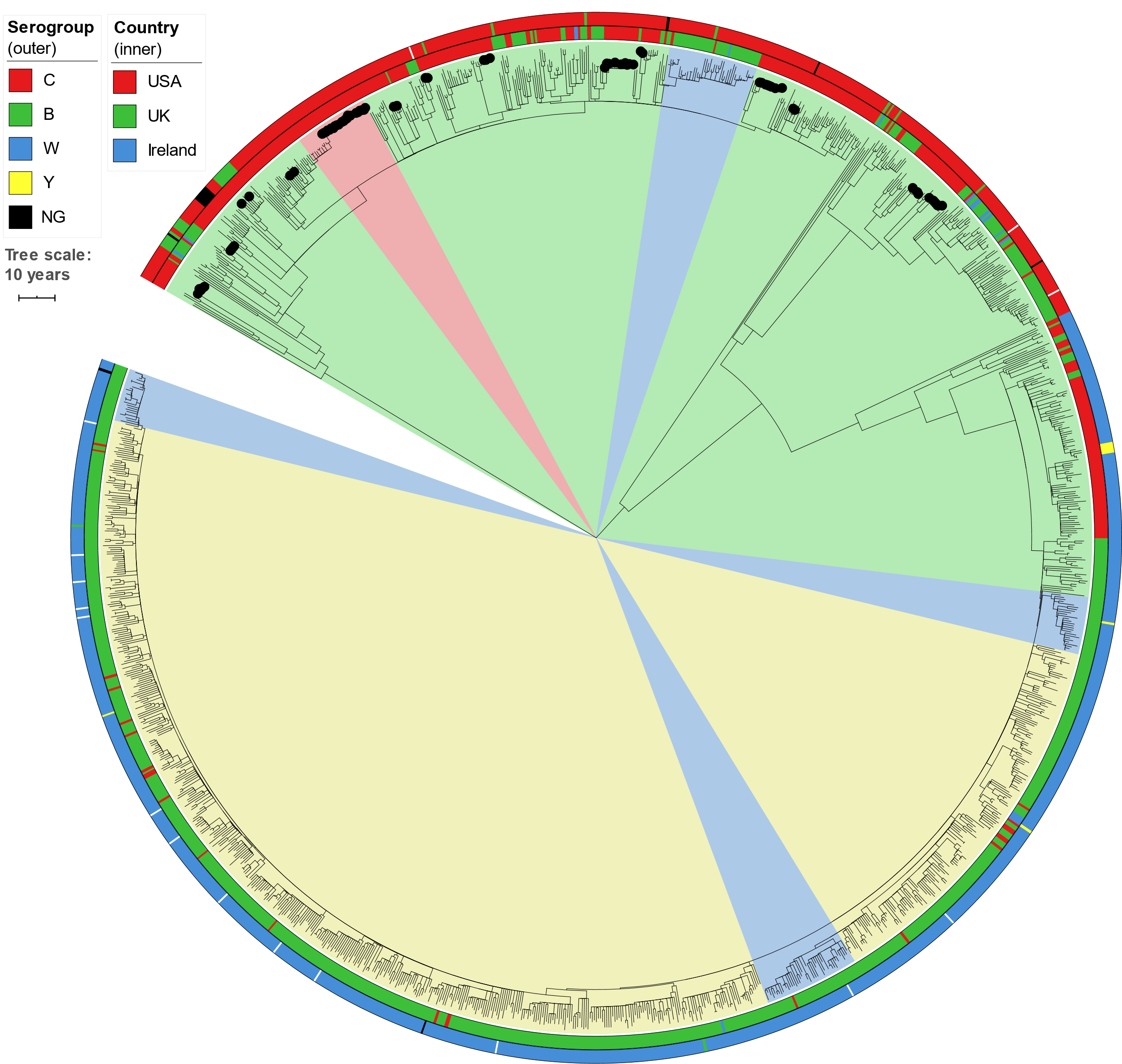


**S3 Fig**: Time-calibrated phylogeny of genomic cluster 1 (CC11, 1423 isolates, 1,160,070bp core genome alignment). The inner ring shows the country of origin and the outer ring shows serogroup (missing data is uncolored). Internal shading shows TreeStructure partitions, with the US-specific partition shaded red. Black dots on branch tips indicate isolates from 14 outbreak clades in the USA. Tree scale bar is 10 years. The estimated evolutionary rate is 9.0×10^-7^ subs/site/year.
