# Supplementary material for: Using Neisseria meningitidis genomic diversity to inform outbreak strain identification
Source: PLoS Pathog. 2021 May 18;17(5):e1009586. doi: 10.1371/journal.ppat.1009586 (PMC8177650; doi:10.1371/journal.ppat.1009586)
Supplement: S5 Fig — Inner ring shows the country of origin, outer ring shows serogroup. Internal shading shows TreeStructure partitions, with the US-specific partition #1 shaded red (detail in Fig 2). Black dots indicate isolates from 11 outbreak clades in the USA. Tree scale bar is 10 years. The estimated evolutionary rate is 9.8×10^−7^ subs/site/year. (DOCX) [file ppat.1009586.s007.docx]

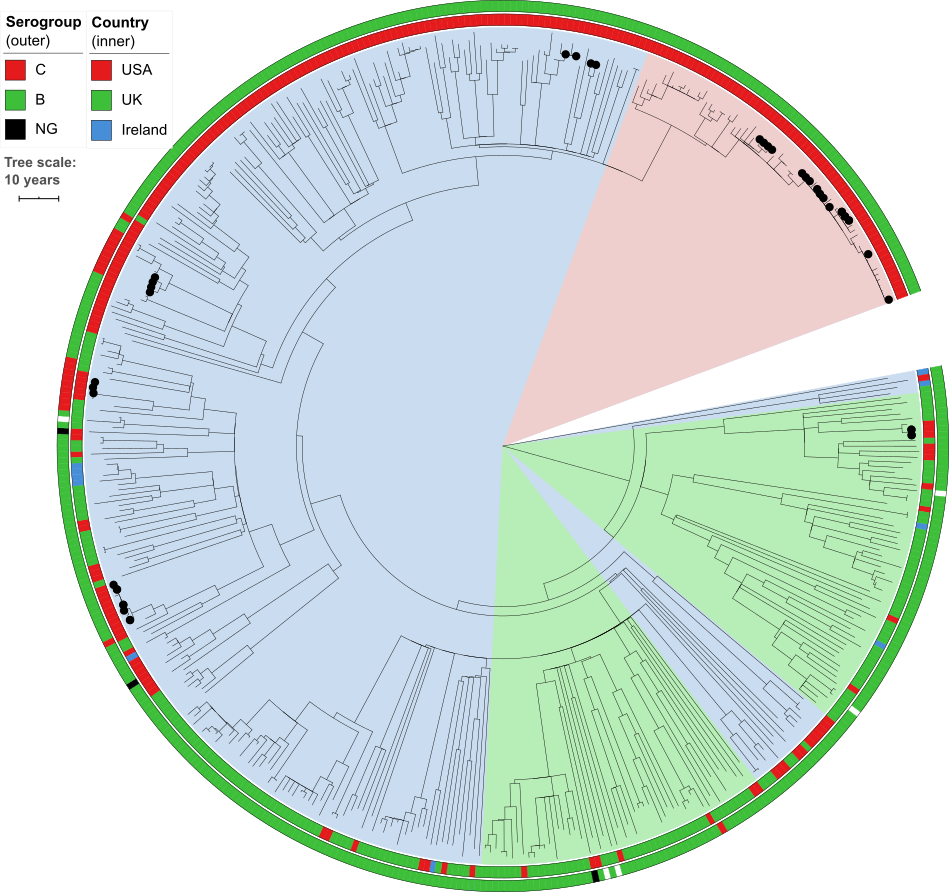


**S5 Fig**: Time-calibrated phylogeny of genomic cluster 5 (CC32, 454 isolates, 1,277,833bp core genome alignment). Inner ring shows the country of origin, outer ring shows serogroup. Internal shading shows TreeStructure partitions, with the US-specific partition #1 shaded red (detail in Fig 2). Black dots indicate isolates from 11 outbreak clades in the USA. Tree scale bar is 10 years. The estimated evolutionary rate is 9.8×10^-7^ subs/site/year.
