# Supplementary material for: Using Neisseria meningitidis genomic diversity to inform outbreak strain identification
Source: PLoS Pathog. 2021 May 18;17(5):e1009586. doi: 10.1371/journal.ppat.1009586 (PMC8177650; doi:10.1371/journal.ppat.1009586)
Supplement: S6 Fig — Inner ring shows the country of origin, outer ring shows serogroup. Internal shading shows TreeStructure partitions: red, partition 1; green, partition 2; blue, partition 3. Black dots indicate isolates from one outbreak clade in the USA. Tree scale bar is 10 years. The estimated evolutionary rate is 1.2×10^−6^ subs/site/year. (DOCX) [file ppat.1009586.s008.docx]

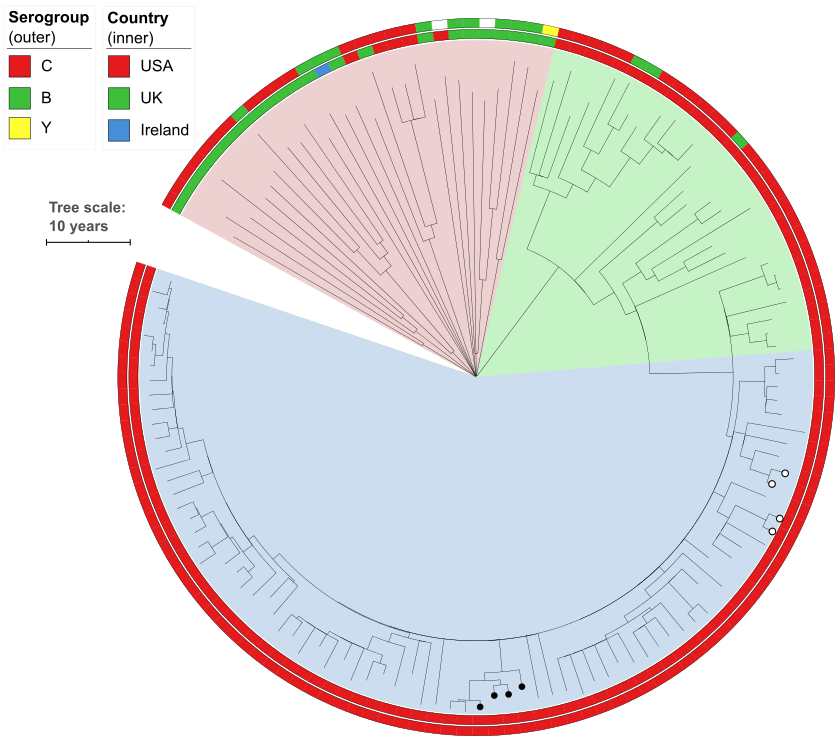


**S6 Fig**: Time-calibrated phylogeny of genomic cluster 8 (CC103, 136 isolates, 1,597,249bp core genome alignment). Inner ring shows the country of origin, outer ring shows serogroup. Internal shading shows TreeStructure partitions: red, partition 1; green, partition 2; blue, partition 3. Black dots indicate isolates from one outbreak clade in the USA; white dots indicate isolates from two pairs of close contacts. Tree scale bar is 10 years. The estimated evolutionary rate is 1.2×10^-6^ subs/site/year.
