# Supplementary material for: Using Neisseria meningitidis genomic diversity to inform outbreak strain identification
Source: PLoS Pathog. 2021 May 18;17(5):e1009586. doi: 10.1371/journal.ppat.1009586 (PMC8177650; doi:10.1371/journal.ppat.1009586)
Supplement: S7 Fig — Inner ring shows the country of origin, outer ring shows serogroup. Black dots indicate isolates from one outbreak clade in the USA. Tree scale bar is 10 years. The estimated evolutionary rate is 1.0×10^−6^ subs/site/year. (DOCX) [file ppat.1009586.s009.docx]

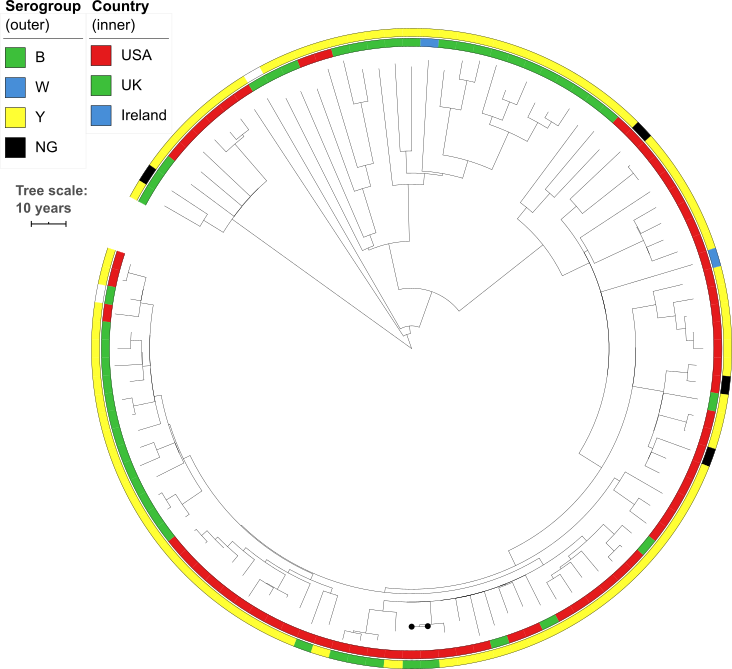


**S7 Fig:** Time-calibrated phylogeny of genomic cluster 9 (CC167, 105 isolates, 1,543,098bp core genome alignment). Inner ring shows the country of origin, outer ring shows serogroup. Black dots indicate isolates from one outbreak clade in the USA. Tree scale bar is 10 years. The estimated evolutionary rate is 1.0×10^-6^ subs/site/year.
