# Supplementary material for: Using Neisseria meningitidis genomic diversity to inform outbreak strain identification
Source: PLoS Pathog. 2021 May 18;17(5):e1009586. doi: 10.1371/journal.ppat.1009586 (PMC8177650; doi:10.1371/journal.ppat.1009586)
Supplement: S8 Fig — Cumulative distribution of alternative distance metrics: kSNP-25 (A), kSNP-251 (B), cgMLST (C). As in Fig 2, distances are categorized based on whether the isolates were collected from the same organization-based outbreak (Org OB: red), community-based outbreak (Com OB: green), or the isolates are not part of a known outbreak, but have the same serogroup and were collected within three months of each other (SG, 3-mo: blue) and from the same state (SG, State, 3-mo: orange). The total number of comparisons in each category is in the parentheses. (DOCX) [file ppat.1009586.s010.docx]

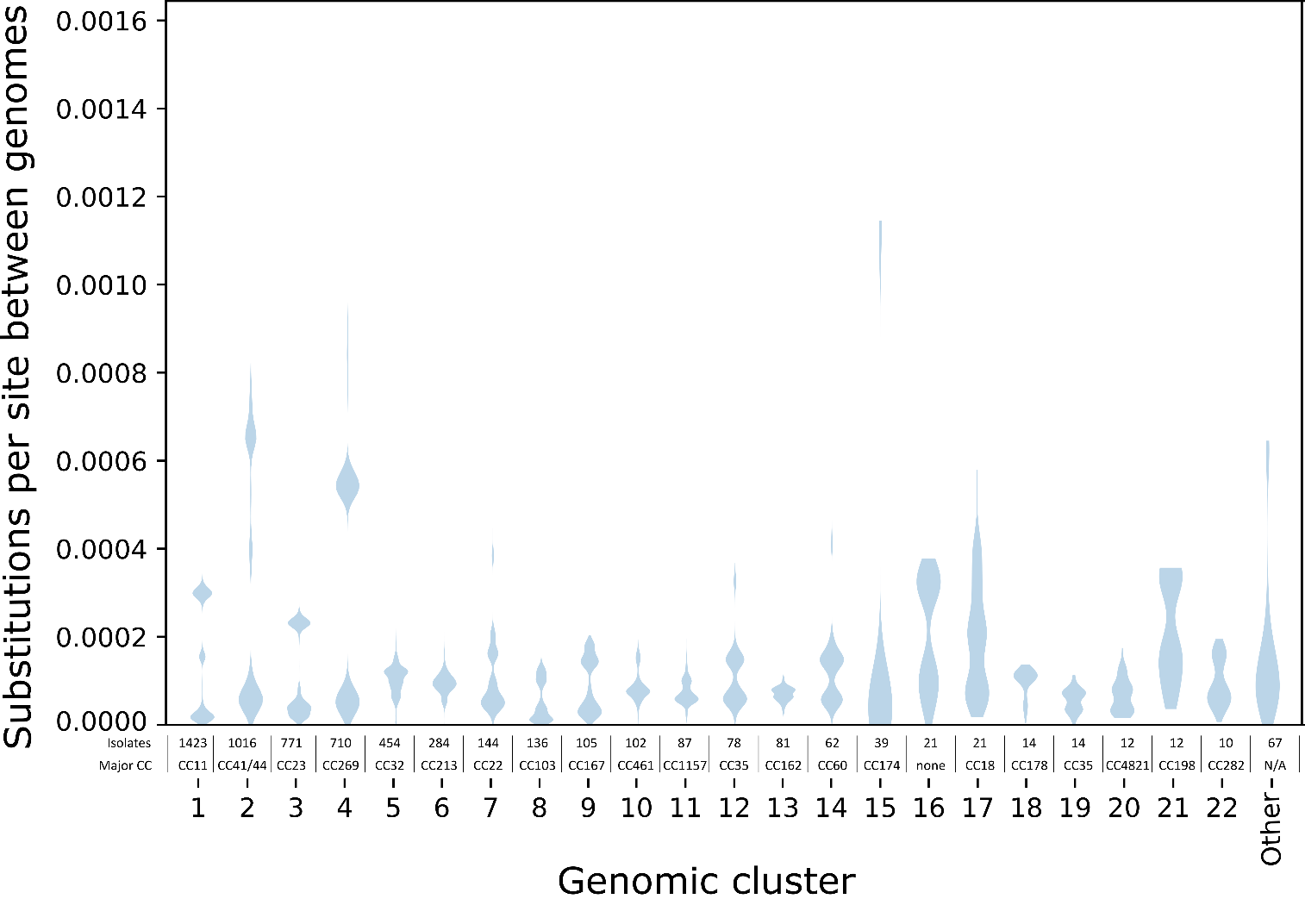


**S1 Fig:** Violin plot showing the distribution of phylogenetic distances within genomic clusters. The 22 genomic clusters with ten or more genomes are labeled with their numeric identifier, while the 10 clusters with five to nine genomes are grouped together as “Other”. The major clonal complexes (CC) and number of isolates in each cluster is written above the numeric identifier. The genomes in cluster 16 had sequence types that were not assigned to any clonal complex (labeled ‘none’), and the major clonal complex was not applicable (“N/A”) to the ‘other’ category because it comprised multiple clusters. Substitutions per site between pairs of genomes were calculated as the sum of branch lengths separating the two genomes on a recombination-corrected maximum likelihood phylogenetic tree that was inferred for each cluster. Phylogenetic trees were not inferred for the 62 genomic clusters with four or fewer genomes in them (including 43 singleton ‘clusters’).


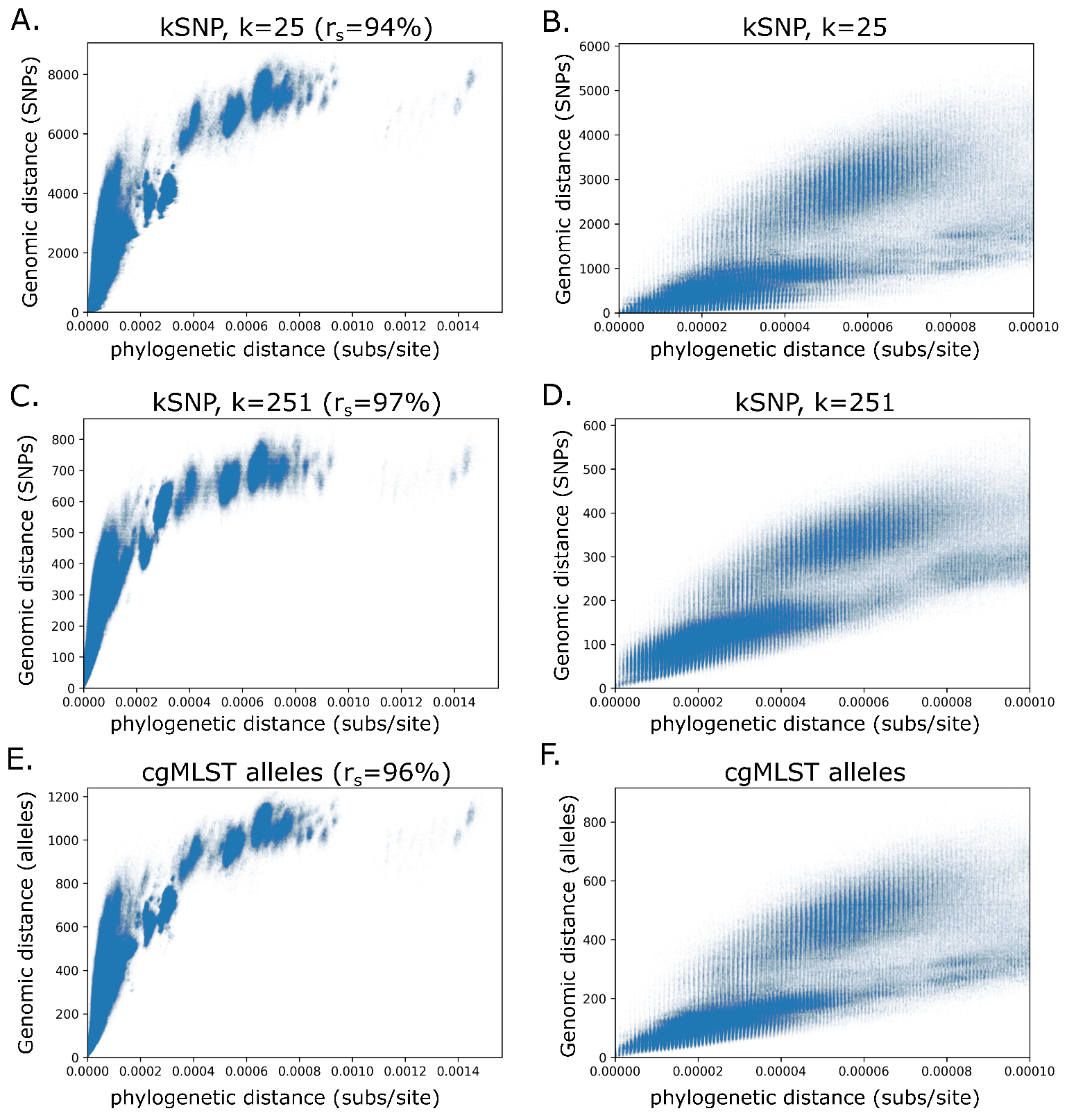


**S2 Fig**: Relationship of phylogenetic distance to other genome distance metrics. The scatter plots on the left (A, C, E) show the full range of distances within genomic clusters and the Spearman rank correlation (*r*_s_), while the scatter plots on the right (B, D, F) are limited to closely related isolate pairs, up to a phylogenetic distance of 10^-4^ subs/site. The distance metrics include SNP distances identified with 25bp k-mer (A, B), SNP distances identified with a 251bp k-mer (C,D), and cgMLST allele distances (E,F).


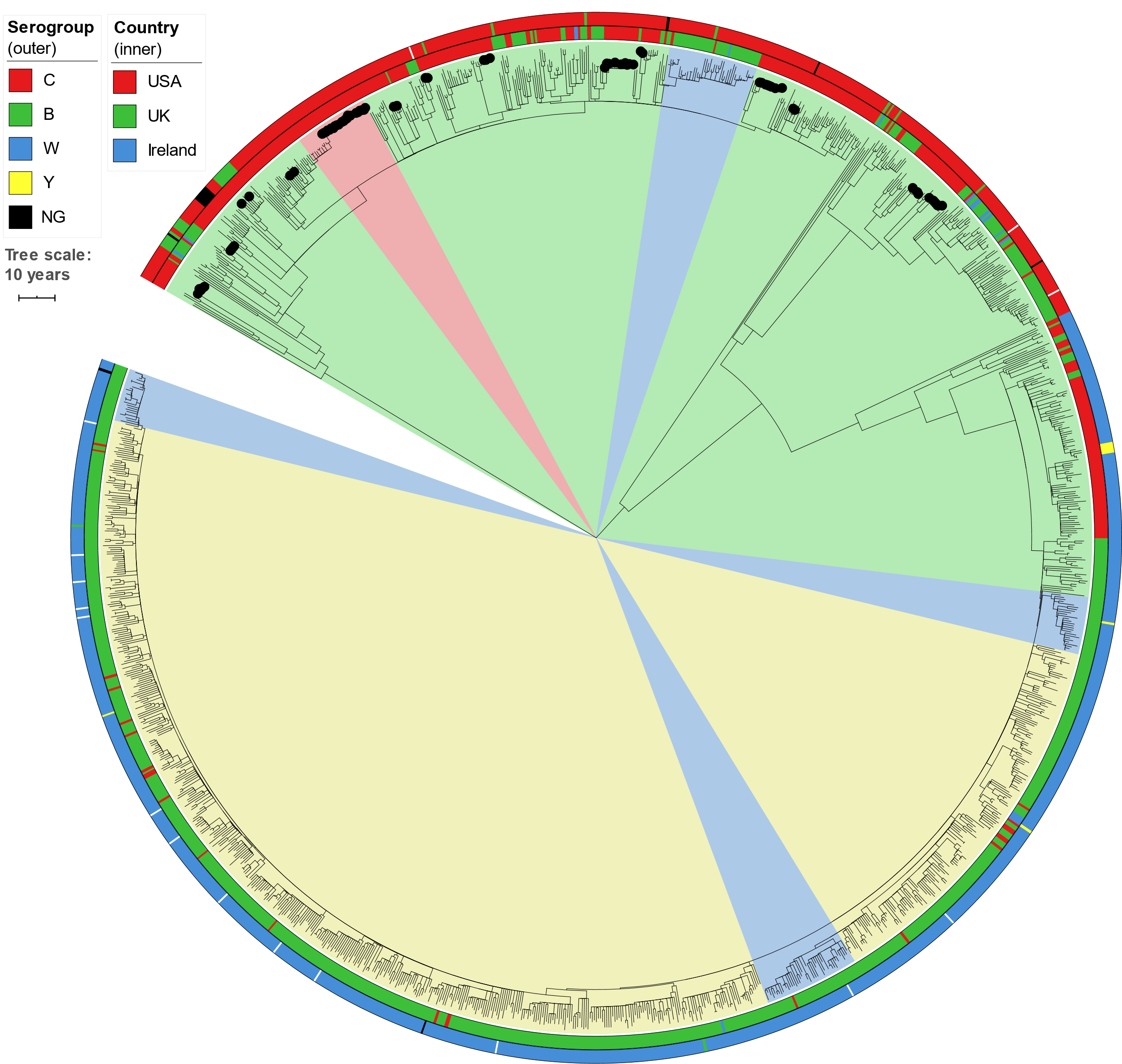


**S3 Fig**: Time-calibrated phylogeny of genomic cluster 1 (CC11, 1423 isolates, 1,160,070bp core genome alignment). The inner ring shows the country of origin and the outer ring shows serogroup (missing data is uncolored). Internal shading shows TreeStructure partitions, with the US-specific partition shaded red. Black dots on branch tips indicate isolates from 14 outbreak clades in the USA. Tree scale bar is 10 years. The estimated evolutionary rate is 9.0×10^-7^ subs/site/year.


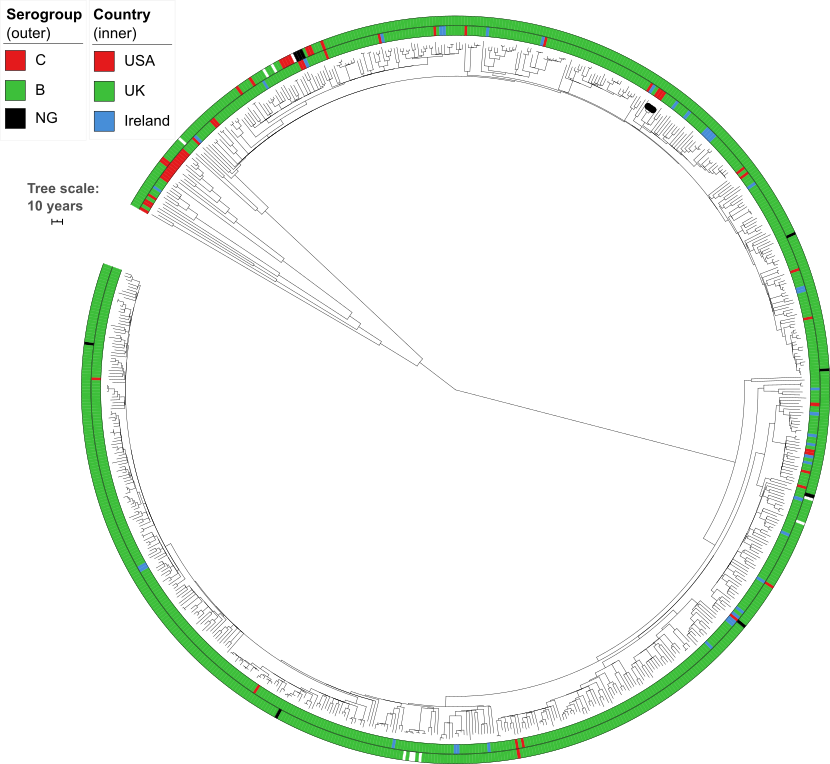


**S4 Fig:** Time-calibrated phylogeny of genomic cluster 4 (CC269, 710 isolates, 1,045,137bp core genome alignment). Inner ring shows the country of origin, outer ring shows serogroup. Black dots on leaf tips indicate isolates from one outbreak clade in the USA. Tree scale bar is 10 years. The estimated evolutionary rate is 8.6×10^-7^ subs/site/year.


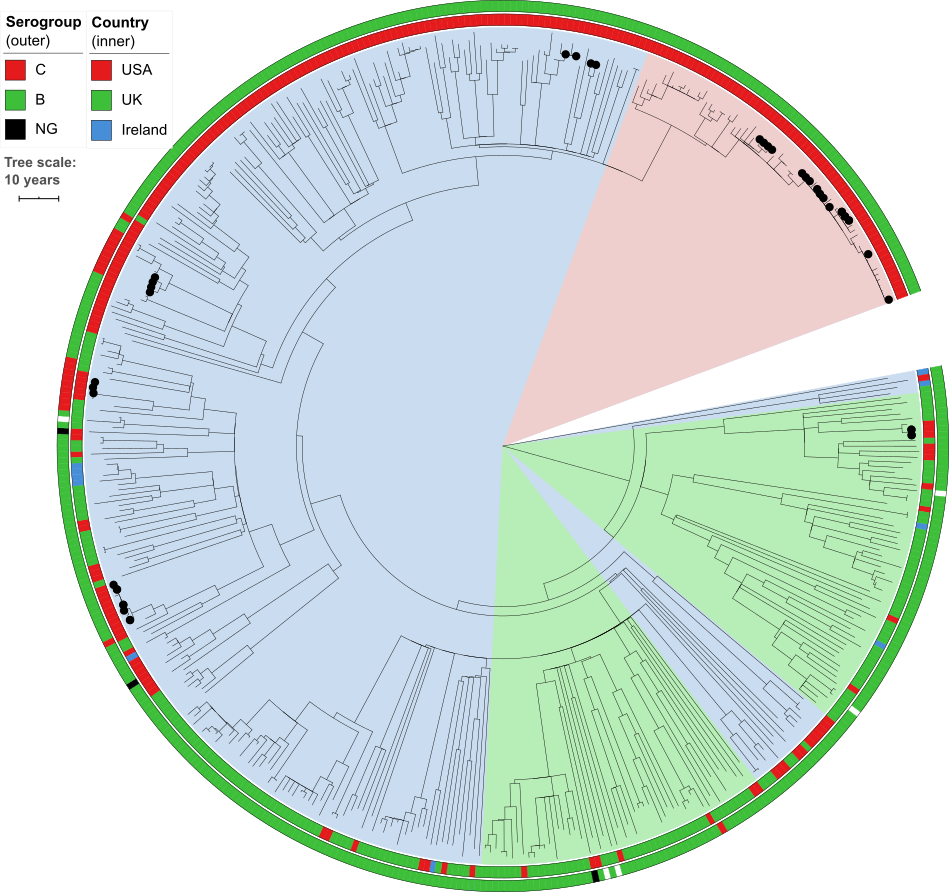


**S5 Fig**: Time-calibrated phylogeny of genomic cluster 5 (CC32, 454 isolates, 1,277,833bp core genome alignment). Inner ring shows the country of origin, outer ring shows serogroup. Internal shading shows TreeStructure partitions, with the US-specific partition #1 shaded red (detail in Fig 2). Black dots indicate isolates from 11 outbreak clades in the USA. Tree scale bar is 10 years. The estimated evolutionary rate is 9.8×10^-7^ subs/site/year.


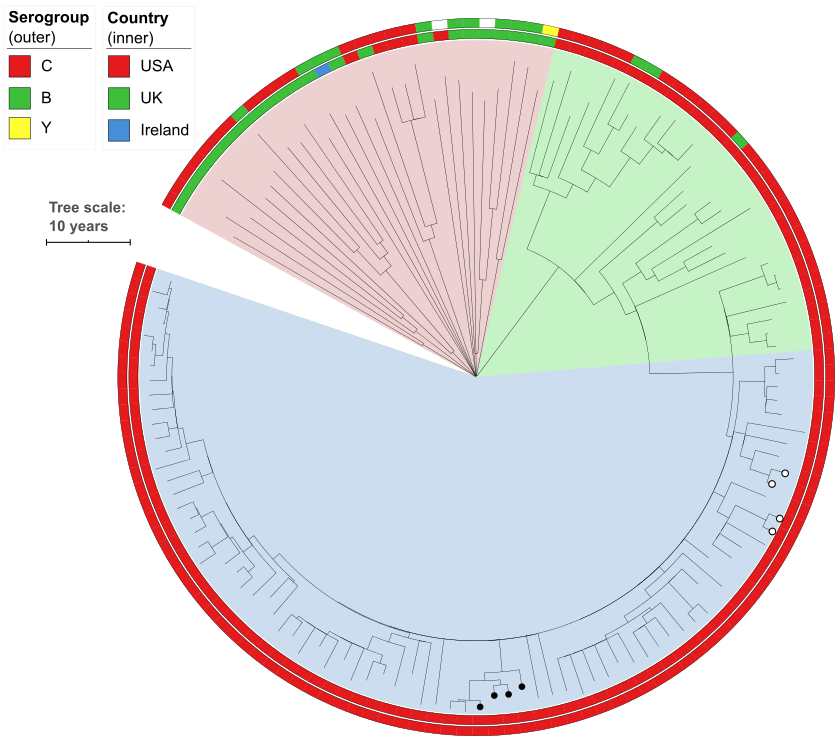


**S6 Fig**: Time-calibrated phylogeny of genomic cluster 8 (CC103, 136 isolates, 1,597,249bp core genome alignment). Inner ring shows the country of origin, outer ring shows serogroup. Internal shading shows TreeStructure partitions: red, partition 1; green, partition 2; blue, partition 3. Black dots indicate isolates from one outbreak clade in the USA; white dots indicate isolates from two pairs of close contacts. Tree scale bar is 10 years. The estimated evolutionary rate is 1.2×10^-6^ subs/site/year.


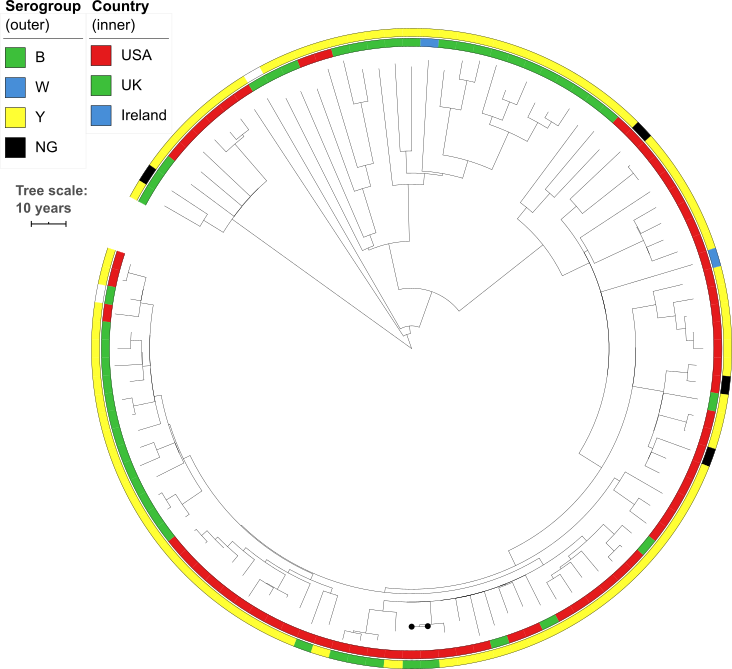


**S7 Fig:** Time-calibrated phylogeny of genomic cluster 9 (CC167, 105 isolates, 1,543,098bp core genome alignment). Inner ring shows the country of origin, outer ring shows serogroup. Black dots indicate isolates from one outbreak clade in the USA. Tree scale bar is 10 years. The estimated evolutionary rate is 1.0×10^-6^ subs/site/year.


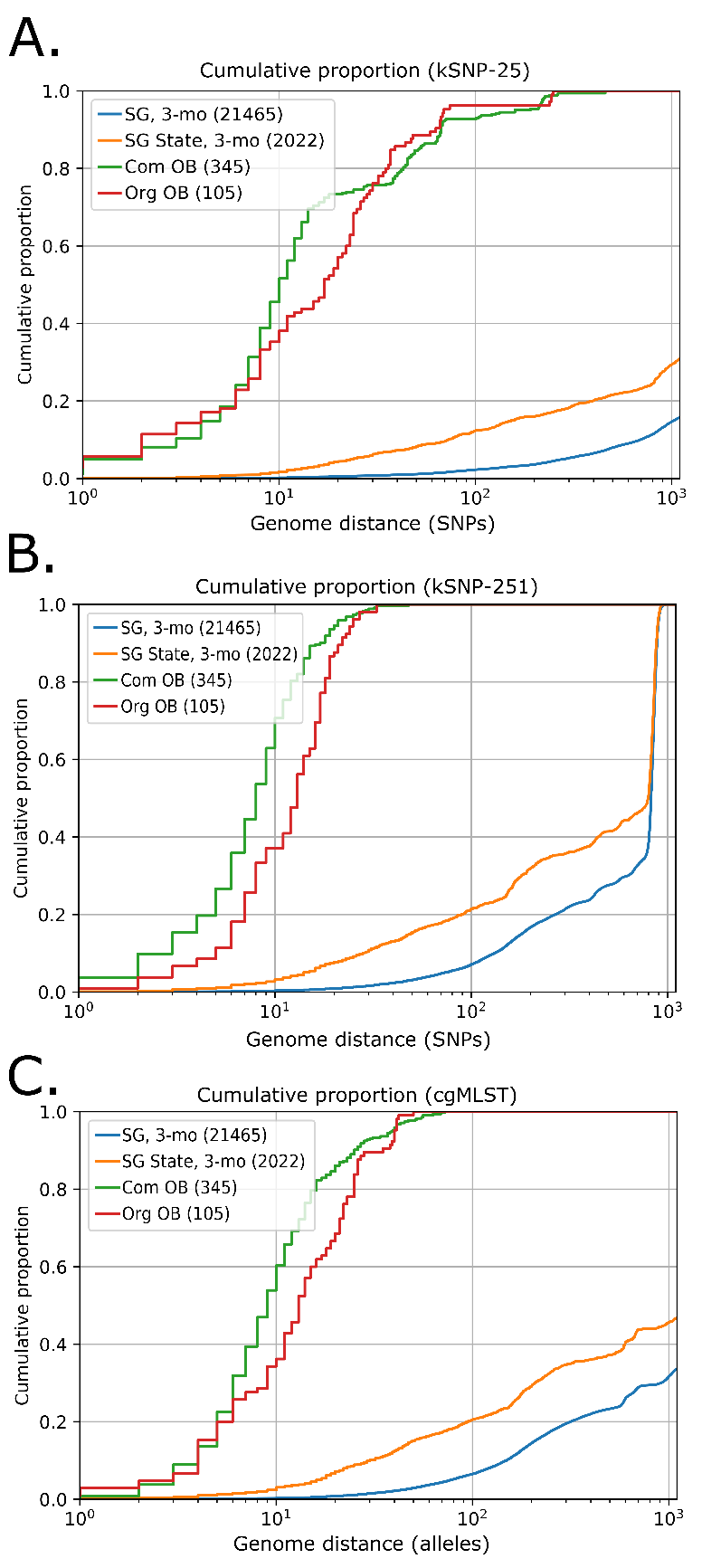


**S8 Fig:** Cumulative distribution of alternative distance metrics: kSNP-25 (A), kSNP-251 (B), cgMLST (C). As in Fig 3, distances are categorized based on whether the isolates were collected from the same organization-based outbreak (Org OB: red), community-based outbreak (Com OB: green), or the isolates are not part of a known outbreak, but have the same serogroup and were collected within three months of each other (SG, 3-mo: blue) and from the same state (SG, State, 3-mo: orange). The total number of comparisons in each category is in the parentheses.


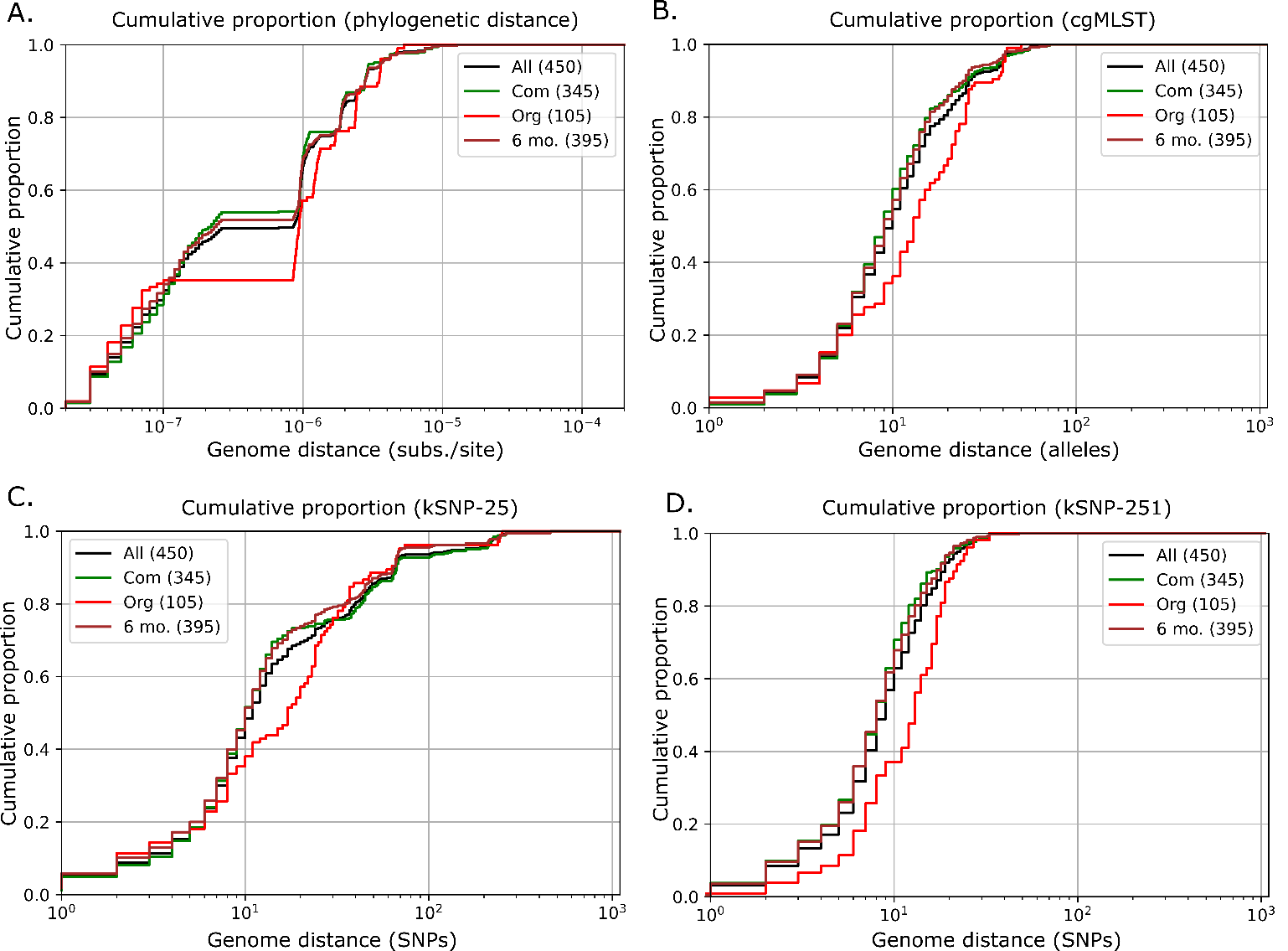


**S9 Fig:** Cumulative distribution of distances among outbreak isolates in the same clade, including a subset that was limited to the pairs that were collected within 6 months of each other. As in Figs 3 and S8, distances are categorized based on whether the isolates were collected from the same organization-based outbreak (Org OB: red), or community-based outbreak (Com OB: green); lines have been added for all outbreaks (black) and comparisons within 6 months (brown).

**
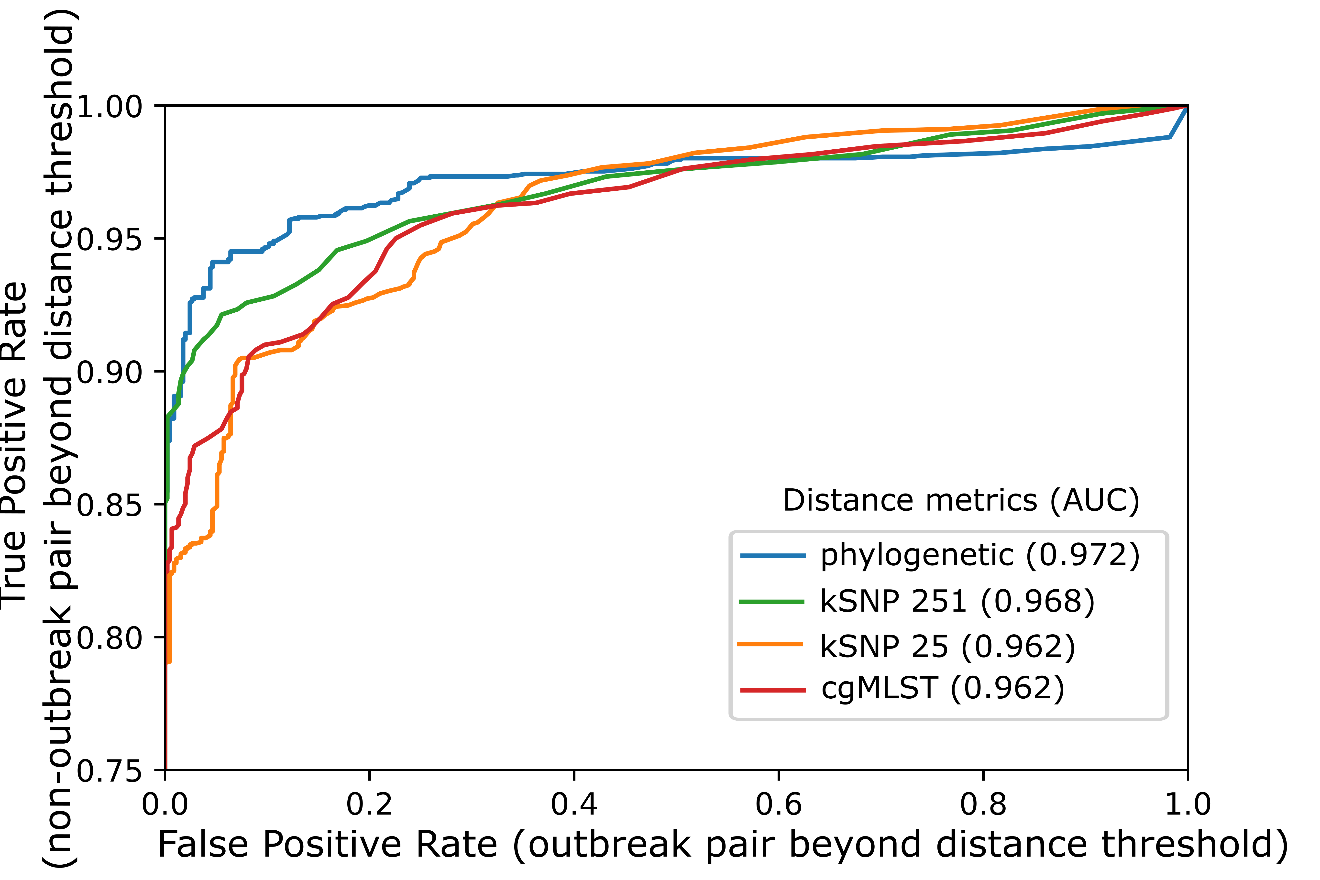
**

**S10 Fig:** ROC (receiver operating characteristic) curves for distinguishing isolate pairs based on each distance metric. AUC (area under the curve) is reported in the legend. A ‘positive’ result occurs when the distance between the two genomes is greater than the threshold value, which decreases as ROC curves move to the top-right. A true positive occurs among the 2,022 pairs of non-outbreak isolates that were the same serogroup and collected in the same state within 92 days of each other; a false positive occurs among the 452 pairs of outbreak isolates that were in the same outbreak clade. The lowest TPR value shown on the vertical axis is 75%; all TPR values were at least 77% (Table 3) while the FPR was 0%. Threshold values for several FPRs are shown in Supplementary Table 2.

S11A Fig.


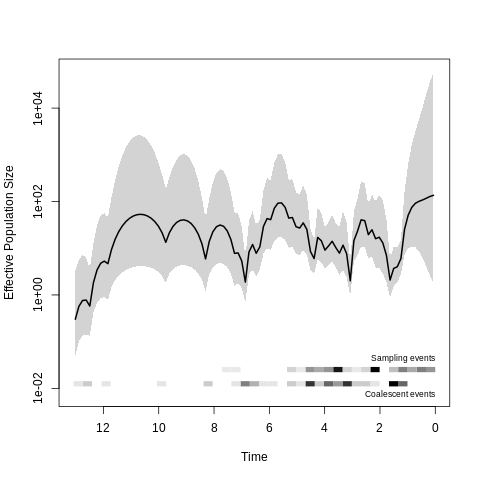


S11B Fig.


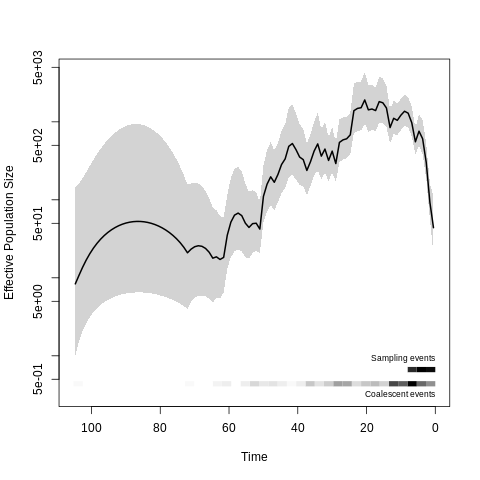


S11C Fig.


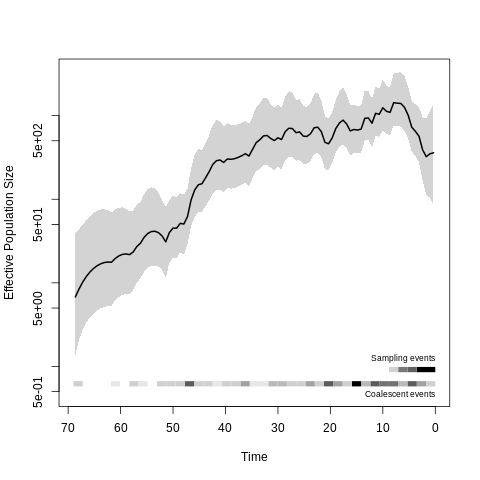


**S11 Fig:** Demographic history of genomic cluster 5 partitions (CC32, shown in S5 Fig). Time is measured in years before 2017, and the effective population size is scaled to the number of generations per year. **A**. Partition 1, consisting of a single clade of 62 US isolates indicated by red shading in supplementary figure 5. **B**. Partition 2 (blue shading). **C**. Partition 3 (green shading).


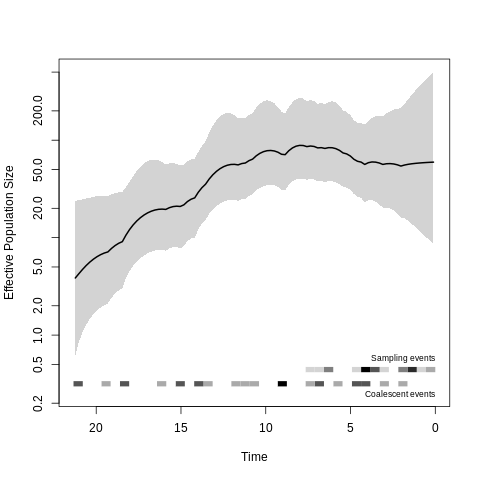
S12A Fig.

S12B Fig.


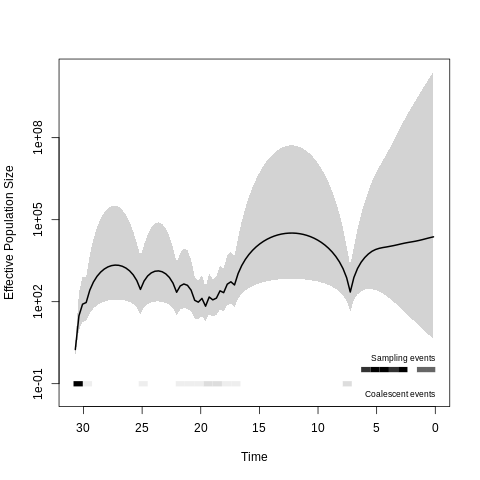


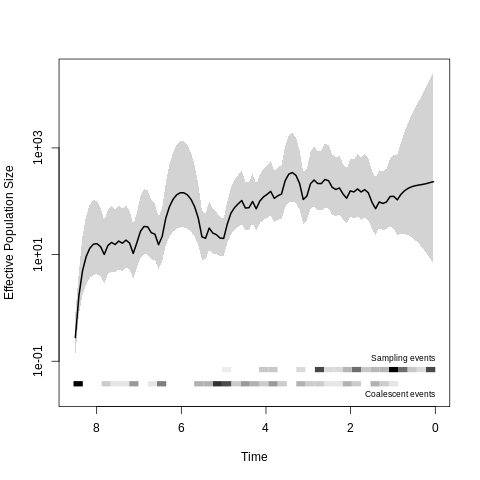
S12C Fig.

**S12 Fig**: Demographic history of cluster 8 partitions (CC103, shown in S6 Fig). Time is measured in years before 2017, and the effective population size is scaled to the number of generations per year. **A**. Partition 1 consisting of 5 US isolates and 24 UK isolates (red shading in supplementary figure 6). **B**. Partition 2, consisting of 27 US isolates (green shading). **C**. Partition 3, consisting of 79 US isolates (blue shading).
