# Supplementary material for: Using Neisseria meningitidis genomic diversity to inform outbreak strain identification
Source: PLoS Pathog. 2021 May 18;17(5):e1009586. doi: 10.1371/journal.ppat.1009586 (PMC8177650; doi:10.1371/journal.ppat.1009586)
Supplement: S9 Fig — As in Figs 3 and S8 distances are categorized based on whether the isolates were collected from the same organization-based outbreak (Org OB: red), or community-based outbreak (Com OB: green); lines have been added for all outbreaks (black) and comparisons within 6 months (brown). (DOCX) [file ppat.1009586.s011.docx]

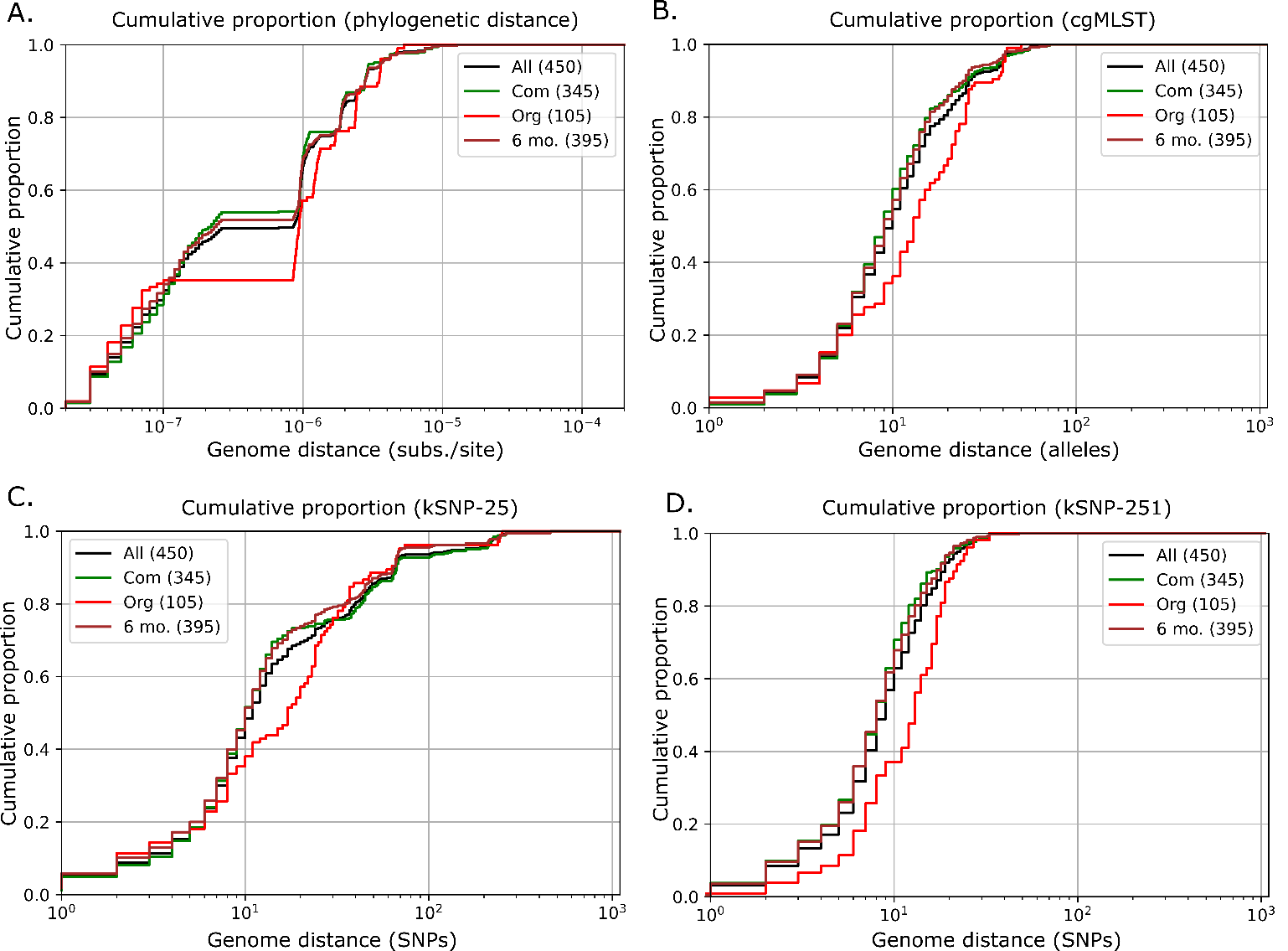


**S9 Fig:** Cumulative distribution of distances among outbreak isolates in the same clade, including a subset that was limited to the pairs that were collected within 6 months of each other. As in Figs 3 and S8, distances are categorized based on whether the isolates were collected from the same organization-based outbreak (Org OB: red), or community-based outbreak (Com OB: green); lines have been added for all outbreaks (black) and comparisons within 6 months (brown).
