# Supplementary material for: Using Neisseria meningitidis genomic diversity to inform outbreak strain identification
Source: PLoS Pathog. 2021 May 18;17(5):e1009586. doi: 10.1371/journal.ppat.1009586 (PMC8177650; doi:10.1371/journal.ppat.1009586)
Supplement: S10 Fig — AUC (area under the curve) is reported in the legend. A ‘positive’ result occurs when the distance between the two genomes is greater than the threshold value, which decreases as ROC curves move to the top-right. A true positive occurs among the 2,022 pairs of non-outbreak isolates that were the same serogroup and collected in the same state within 92 days of each other; a false positive occurs among the 452 pairs of outbreak isolates that were in the same outbreak clade. The lowest TPR value shown on the vertical axis is 75%; all TPR values were at least 77% (Table 3) while the FPR was 0%. Threshold values for several FPRs are shown in S2 Table. (DOCX) [file ppat.1009586.s012.docx]

**
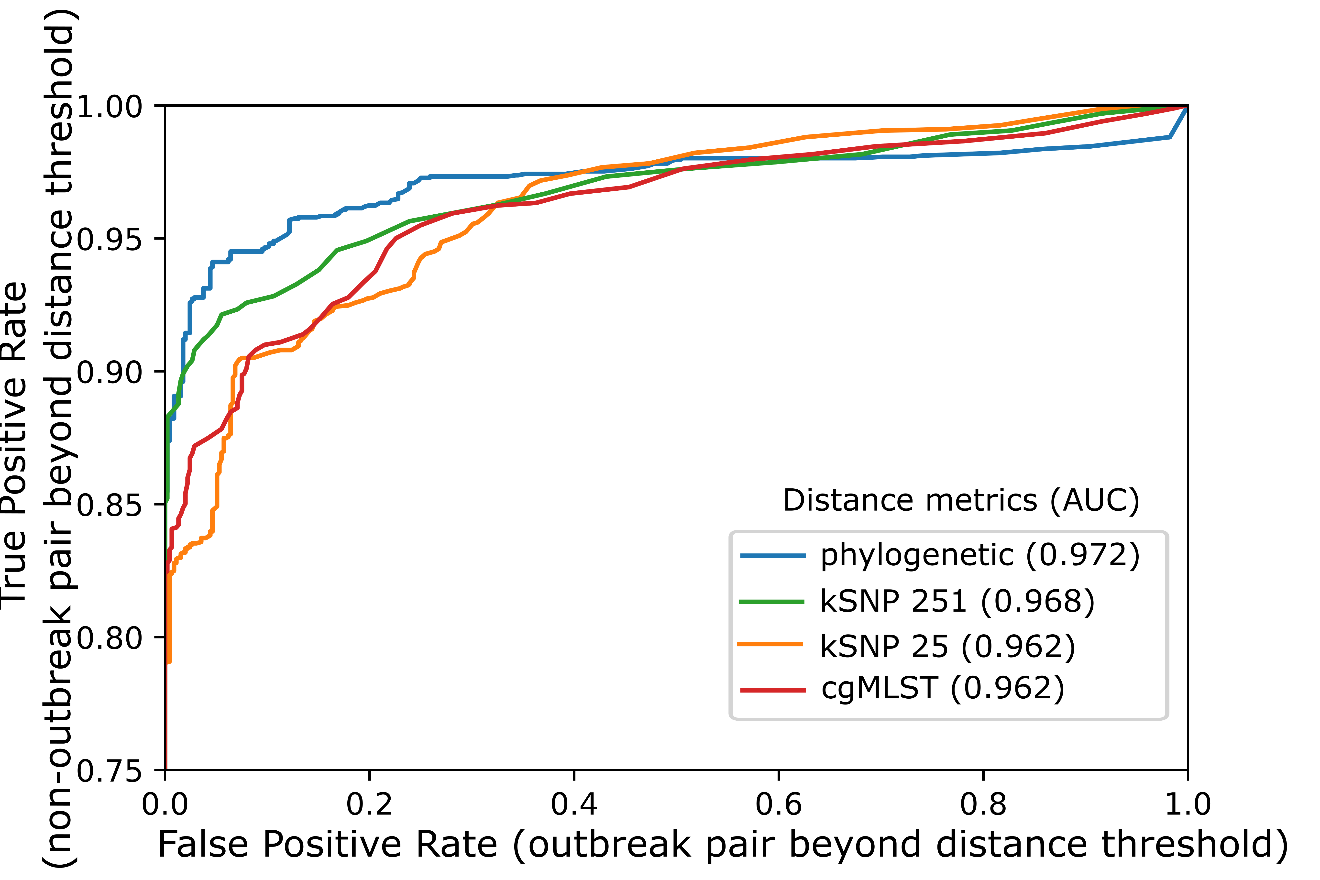
**

**S10 Fig:** ROC (receiver operating characteristic) curves for distinguishing isolate pairs based on each distance metric. AUC (area under the curve) is reported in the legend. A ‘positive’ result occurs when the distance between the two genomes is greater than the threshold value, which decreases as ROC curves move to the top-right. A true positive occurs among the 2,022 pairs of non-outbreak isolates that were the same serogroup and collected in the same state within 92 days of each other; a false positive occurs among the 452 pairs of outbreak isolates that were in the same outbreak clade. The lowest TPR value shown on the vertical axis is 75%; all TPR values were at least 77% (Table 3) while the FPR was 0%. Threshold values for several FPRs are shown in Supplementary Table 2.
