# Supplementary material for: Using Neisseria meningitidis genomic diversity to inform outbreak strain identification
Source: PLoS Pathog. 2021 May 18;17(5):e1009586. doi: 10.1371/journal.ppat.1009586 (PMC8177650; doi:10.1371/journal.ppat.1009586)
Supplement: S11 Fig — Time is measured in years before 2017, and the effective population size is scaled to the number of generations per year. A. Partition 1, consisting of a single clade of 62 US isolates indicated by red shading in S5 Fig. B. Partition 2 (blue shading). C. Partition 3 (green shading). (DOCX) [file ppat.1009586.s013.docx]

S11A Fig.


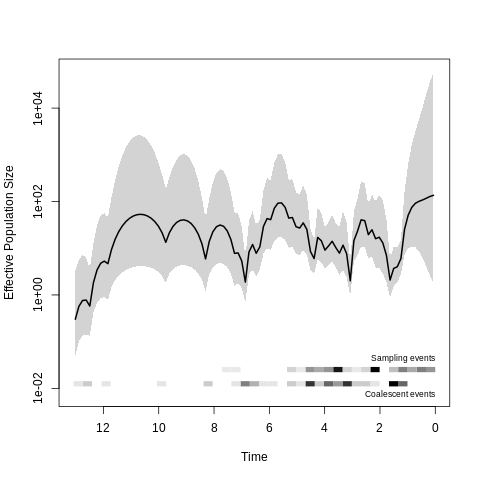


S11B Fig.


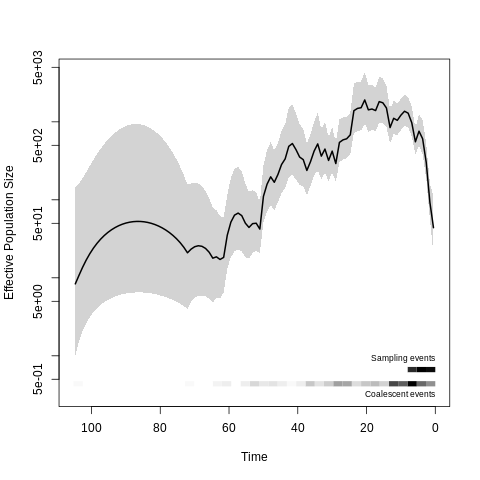


S11C Fig.


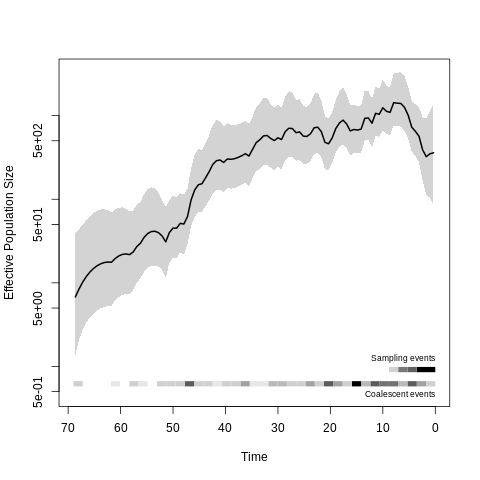


**S11 Fig:** Demographic history of genomic cluster 5 partitions (CC32, shown in S5 Fig). Time is measured in years before 2017, and the effective population size is scaled to the number of generations per year. **A**. Partition 1, consisting of a single clade of 62 US isolates indicated by red shading in supplementary figure 5. **B**. Partition 2 (blue shading). **C**. Partition 3 (green shading).
