# Supplementary material for: Using Neisseria meningitidis genomic diversity to inform outbreak strain identification
Source: PLoS Pathog. 2021 May 18;17(5):e1009586. doi: 10.1371/journal.ppat.1009586 (PMC8177650; doi:10.1371/journal.ppat.1009586)
Supplement: S12 Fig — Time is measured in years before 2017, and the effective population size is scaled to the number of generations per year. A. Partition 1 consisting of 5 US isolates and 24 UK isolates (red shading in S6 Fig). B. Partition 2, consisting of 27 US isolates (green shading). C. Partition 3, consisting of 79 US isolates (blue shading). (DOCX) [file ppat.1009586.s014.docx]

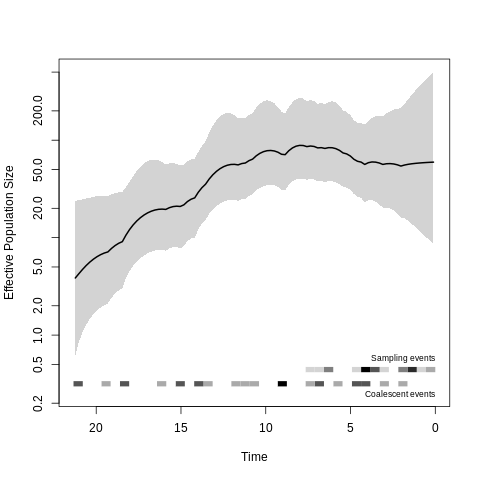
S12A Fig.

S12B Fig.


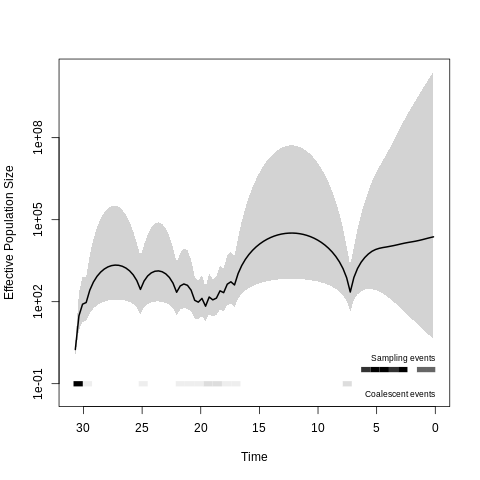


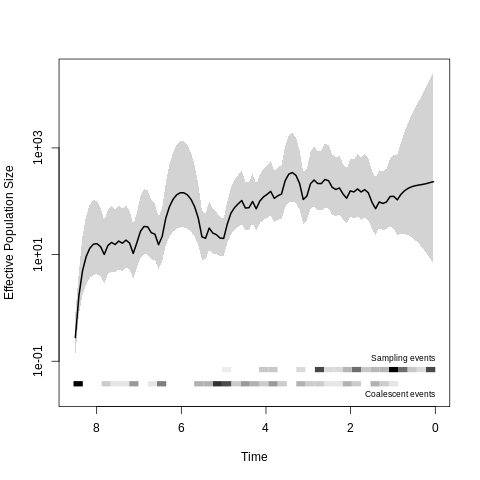
S12C Fig.

**S12 Fig**: Demographic history of cluster 8 partitions (CC103, shown in S6 Fig). Time is measured in years before 2017, and the effective population size is scaled to the number of generations per year. **A**. Partition 1 consisting of 5 US isolates and 24 UK isolates (red shading in supplementary figure 6). **B**. Partition 2, consisting of 27 US isolates (green shading). **C**. Partition 3, consisting of 79 US isolates (blue shading).
